# Comprehension of acoustically degraded speech in Alzheimer’s disease and primary progressive aphasia

**DOI:** 10.1093/brain/awad163

**Published:** 2023-05-15

**Authors:** Jessica Jiang, Jeremy C S Johnson, Maï-Carmen Requena-Komuro, Elia Benhamou, Harri Sivasathiaseelan, Anthipa Chokesuwattanaskul, Annabel Nelson, Ross Nortley, Rimona S Weil, Anna Volkmer, Charles R Marshall, Doris-Eva Bamiou, Jason D Warren, Chris J D Hardy

**Affiliations:** Dementia Research Centre, Department of Neurodegenerative Disease, UCL Queen Square Institute of Neurology, University College London, London WC1N 3AR, UK; Dementia Research Centre, Department of Neurodegenerative Disease, UCL Queen Square Institute of Neurology, University College London, London WC1N 3AR, UK; Dementia Research Centre, Department of Neurodegenerative Disease, UCL Queen Square Institute of Neurology, University College London, London WC1N 3AR, UK; Kidney Cancer Program, UT Southwestern Medical Centre, Dallas, TX 75390, USA; Dementia Research Centre, Department of Neurodegenerative Disease, UCL Queen Square Institute of Neurology, University College London, London WC1N 3AR, UK; Dementia Research Centre, Department of Neurodegenerative Disease, UCL Queen Square Institute of Neurology, University College London, London WC1N 3AR, UK; Dementia Research Centre, Department of Neurodegenerative Disease, UCL Queen Square Institute of Neurology, University College London, London WC1N 3AR, UK; Division of Neurology, Department of Internal Medicine, King Chulalongkorn Memorial Hospital, Thai Red Cross Society, Bangkok 10330, Thailand; Dementia Research Centre, Department of Neurodegenerative Disease, UCL Queen Square Institute of Neurology, University College London, London WC1N 3AR, UK; Dementia Research Centre, Department of Neurodegenerative Disease, UCL Queen Square Institute of Neurology, University College London, London WC1N 3AR, UK; Wexham Park Hospital, Frimley Health NHS Foundation Trust, Slough SL2 4HL, UK; Dementia Research Centre, Department of Neurodegenerative Disease, UCL Queen Square Institute of Neurology, University College London, London WC1N 3AR, UK; Division of Psychology and Language Sciences, University College London, London WC1H 0AP, UK; Preventive Neurology Unit, Wolfson Institute of Population Health, Queen Mary University of London, London EC1M 6BQ, UK; UCL Ear Institute and UCL/UCLH Biomedical Research Centre, National Institute of Health Research, University College London, London WC1X 8EE, UK; Dementia Research Centre, Department of Neurodegenerative Disease, UCL Queen Square Institute of Neurology, University College London, London WC1N 3AR, UK; Dementia Research Centre, Department of Neurodegenerative Disease, UCL Queen Square Institute of Neurology, University College London, London WC1N 3AR, UK

**Keywords:** degraded speech, auditory perception, primary progressive aphasia, frontotemporal dementia, Alzheimer’s disease

## Abstract

Successful communication in daily life depends on accurate decoding of speech signals that are acoustically degraded by challenging listening conditions. This process presents the brain with a demanding computational task that is vulnerable to neurodegenerative pathologies. However, despite recent intense interest in the link between hearing impairment and dementia, comprehension of acoustically degraded speech in these diseases has been little studied. Here we addressed this issue in a cohort of 19 patients with typical Alzheimer’s disease and 30 patients representing the three canonical syndromes of primary progressive aphasia (non-fluent/agrammatic variant primary progressive aphasia; semantic variant primary progressive aphasia; logopenic variant primary progressive aphasia), compared to 25 healthy age-matched controls. As a paradigm for the acoustically degraded speech signals of daily life, we used noise-vocoding: synthetic division of the speech signal into frequency channels constituted from amplitude-modulated white noise, such that fewer channels convey less spectrotemporal detail thereby reducing intelligibility.

We investigated the impact of noise-vocoding on recognition of spoken three-digit numbers and used psychometric modelling to ascertain the threshold number of noise-vocoding channels required for 50% intelligibility by each participant. Associations of noise-vocoded speech intelligibility threshold with general demographic, clinical and neuropsychological characteristics and regional grey matter volume (defined by voxel-based morphometry of patients’ brain images) were also assessed. Mean noise-vocoded speech intelligibility threshold was significantly higher in all patient groups than healthy controls, and significantly higher in Alzheimer’s disease and logopenic variant primary progressive aphasia than semantic variant primary progressive aphasia (all *P* < 0.05). In a receiver operating characteristic analysis, vocoded intelligibility threshold discriminated Alzheimer’s disease, non-fluent variant and logopenic variant primary progressive aphasia patients very well from healthy controls. Further, this central hearing measure correlated with overall disease severity but not with peripheral hearing or clear speech perception. Neuroanatomically, after correcting for multiple voxel-wise comparisons in predefined regions of interest, impaired noise-vocoded speech comprehension across syndromes was significantly associated (*P* < 0.05) with atrophy of left planum temporale, angular gyrus and anterior cingulate gyrus: a cortical network that has previously been widely implicated in processing degraded speech signals.

Our findings suggest that the comprehension of acoustically altered speech captures an auditory brain process relevant to daily hearing and communication in major dementia syndromes, with novel diagnostic and therapeutic implications.

## Teaser

Jiang *et al.* show that people with Alzheimer’s disease and progressive aphasia are impaired versus healthy controls on a degraded speech comprehension task. Using voxel-based morphometry, they show that impaired task performance correlates with atrophy of a fronto-temporal cortical network previously implicated in degraded speech processing.

## Introduction

Successful communication in the world at large depends on our ability to understand spoken messages under non-ideal listening conditions. In our daily lives, we are required to interpret speech that is acoustically degraded by a wide variety of different ways—we regularly conduct conversations over background noise, adapt to suboptimal telephone and video connections and interpret unfamiliar accents. The processing of such degraded speech signals presents the brain with a challenging computational problem, whereby acoustic signals (or ‘auditory objects’) of interest must be disambiguated from interfering (and changing) noise.^1–3^ Because speech signals are critical for communication, decoding of degraded speech is generally the most functionally relevant index of hearing ability in daily life. This process, normally automatic and relatively effortless, is impaired in neurodegenerative disorders such as Alzheimer’s disease and the ‘language-led’ dementia syndromes of the primary progressive aphasia (PPA) spectrum.^4–8^

Hearing impairment has recently been identified as a major risk factor for dementia and a driver of cognitive decline and disability.^4,9,10^ While most studies addressing this linkage have focused on peripheral hearing function measured using the detection of pure tones,^4,11,12^ mounting evidence suggests that measures of central hearing (auditory brain) function and in particular, the comprehension of degraded speech signals, may be more pertinent.^6,8,13,14^ Large cohort studies have identified impaired comprehension of degraded messages as a harbinger of dementia.^7,15,16^ More specifically, Alzheimer’s disease has been shown to impact speech-in-noise perception^17^ and identification of dichotic digits.^6,18–20^ This is likely to reflect, at least in part, a generic impairment of auditory scene analysis in Alzheimer’s disease, affecting the parsing of non-verbal as well as verbal information and linked to degeneration of the core temporo-parietal ‘default mode’ network targeted by Alzheimer’s disease pathology.^17,21–24^

Further, both Alzheimer’s disease and PPA syndromes impair comprehension of non-native accents,^25–28^ sinewave speech^29,30^ and noise-interrupted speech,^31^ suggesting that neurodegenerative pathologies impair the processing of degraded speech signals more generally. However, the neural mechanisms responsible, the types of speech degradation that are implicated in everyday listening and the effects of different neurodegenerative pathologies have not yet been fully clarified. There are several grounds on which the processing of degraded speech may be especially vulnerable to neurodegenerative pathologies.^5^ Neuroanatomically, the processing of degraded speech signals engages distributed neural networks in perisylvian, prefrontal and posterior temporo-parietal cortices: these same brain networks are targeted preferentially in PPA, particularly the non-fluent/agrammatic variant and logopenic variant syndromes.^5,29,32,33^ Computationally, the comprehension of degraded speech signals depends on precise, yet dynamic integration of information across neural circuitry^4,5,8,34,35^ and neurodegenerative pathologies are likely to blight these computations early and profoundly.

One widely used technique for altering speech signals experimentally is noise-vocoding, whereby a speech signal is divided digitally into discrete frequency bands (‘channels’), each filled with white noise and modulated by the amplitude envelope of the original signal.^36^ This procedure degrades the spectral content of the speech signal while preserving its overall longer range temporal structure. The level of intelligibility of the noise-vocoded speech signal can be controlled parametrically: fewer channels is equivalent to less spectral detail available, leading to less intelligible speech. Noise-vocoding simulates the acoustic characteristics of a cochlear implant, and noise-vocoded speech *per* se will not be encountered by most listeners in everyday life. However, among various alternative methods,^5^ noise-vocoding has certain attributes that make it attractive as a model paradigm to study the effects of disease on the processing of degraded speech more generally.

Noise-vocoding has been widely studied and its behavioural and neuroanatomical correlates in the healthy human brain are fairly well established.^36–42^ As an exemplar of acoustic degradation based on reduction of spectral information, it is likely to capture auditory brain processes engaged by a variety of daily listening scenarios that require decoding of ‘noisy’ speech signals (for example, a poor telephone or video-conferencing line, or a speaker with a heavy cold). In contrast to speech-in-noise techniques (which mix a speech signal with extraneous background sound), noise-vocoding degrades the intrinsic features of the speech signal. It therefore opens a window on auditory perceptual and cognitive processes complementary to those engaged in processing sound scenes (following a speech signal against competing background noise). Comprehension of noise-vocoded speech is likely to be more dependent on auditory object (phonemic) decoding than selective attention: indeed, perceptual and electrophysiological processing of noise-vocoded speech and acoustically degraded conspecific call sounds has been demonstrated in non-human primates,^43–47^ suggesting that noise-vocoding may engage a fundamental neural integrative mechanism for decoding vocal signals in primate auditory cortex. Further, noise-vocoding offers the substantial advantage of generating a quantifiable threshold for intelligibility of the degraded speech signal, based on the number of vocoding channels. This potentially allows for a more sensitive, graded and robust determination of deficit, enabling comparisons between diseases, tracking of disease evolution and potentially, assessing the impact of therapeutic interventions.

Noise-vocoding has been previously applied in a joint behavioural and magnetoencephalographic (MEG) study of non-fluent/agrammatic variant PPA (nfvPPA), to assess the brain mechanisms that mediate comprehension of degraded speech in the context of relatively focal cerebral atrophy.^48^ This work showed that patients with nfvPPA rely more on cross-modal cues to disambiguate vocoded speech signals, and have inflexible predictive decoding mechanisms, instantiated in left inferior frontal cortex. However, noise-vocoding has not been exploited as a tool to compare degraded speech perception in different neurodegenerative syndromes. More generally, the cognitive and neuroanatomical mechanisms that mediate the processing of degraded speech and their clinical relevance in this disease spectrum remain poorly defined.

Here, using noise-vocoding, we evaluated the comprehension of acoustically degraded spoken messages in cohorts of patients with typical Alzheimer’s disease and with all major syndromes of PPA, referenced to healthy older listeners. We assessed how the understanding of noise-vocoded speech was related to other demographic and disease characteristics. We further assessed the structural neuroanatomical associations of the noise-vocoded speech intelligibility threshold in Alzheimer’s disease and PPA, using voxel-based morphometry (VBM) on patients’ brain magnetic resonance images. Based on available evidence with noise-vocoded^48^ and other degraded speech stimuli (e.g. speech-in-noise^16^ and sinewave speech^29^) in Alzheimer’s disease and PPA patients, we hypothesized that both Alzheimer’s disease and PPA patients would have elevated thresholds for comprehending noise-vocoded speech compared with healthy controls, and that this deficit would be more severe in nfvPPA and logopenic variant PPA (lvPPA) than in other neurodegenerative syndromes. We further hypothesized that elevated noise-vocoded intelligibility threshold (as an index of impaired comprehension of degraded speech) would be correlated over the combined patient cohort with regional grey matter atrophy in left posterior superior temporal, inferior parietal and inferior frontal cortices: a network of brain areas previously implicated in the processing of noise-vocoded speech in the healthy brain^36–42^ and targeted early and relatively selectively by neurodegenerative pathology in Alzheimer’s disease and PPA.^49^

## Materials and methods

### Participants

Nineteen patients with typical amnestic Alzheimer’s disease, eight patients with lvPPA, 10 patients with nfvPPA and 12 patients with semantic variant PPA (svPPA) were recruited via a specialist cognitive clinic. All patients fulfilled consensus clinical diagnostic criteria with compatible brain MRI profiles and had clinically mild-to-moderate disease.^50,51^ No patients with pathogenic mutations were included.

Twenty-five healthy older control participants with no history of neurological or psychiatric disorders were recruited from the Dementia Research Centre volunteer database. All participants had a comprehensive general neuropsychological assessment (Table 1). None had a history of otological disease, other than presbycusis; participants assessed in person at the research centre had pure tone audiometry, following a previously described procedure (details in Supplementary material).

**Table 1:** General demographic, clinical and neuropsychological characteristics of all participant groups

Characteristic	Controls	AD	lvPPA	nfvPPA	svPPA
**Demographic and clinical**					
Male: female, *n*	14:11	15:4	7:1	8:2	7:5
Age, years	68.28 (6.62)	70.11 (8.43)*	71.8 (5.50)*	72.7 (3.65)*	63.08 (8.38)
Handedness (R/L/A)	21/1/1^a^	18/1/0	8/0/0	10/0/0	11/1/0
Education (y)	16.13 (2.72)	15.44 (3.79)	14.40 (2.88)	15.10 (2.64)	15.55 (2.07)
Symptom duration (y)	NA	5.94 (3.02)	5.57 (4.86)	3.30 (1.16)	5.42 (2.50)
Best ear average^b^	17.10 (8.72)^c^	27.67 (10.92)^d^	19.00 (10.7)^e^	29.25 (3.30)^e^	23.75 (8.07)^f^
Tested in-person/remote	21/4	10/9	3/5	4/6	8/4
MMSE (/30)	29.75 (0.62)^g^	**20.43 (7.81)**	**22.67 (7.51)^h^**	26.50 (0.71)^a^	**22.88 (5.14)**
T-MMSE (/27)	26.11 (1.76)	**17.75 (4.45)**	**21.50 (4.51)**	24.33 (2.25)	24.00 (1.41)
Percentage of participants taking donepezil and/or memantine	NA	81.25%^i^	83.33%^a^	16.67%^f^	0%^h^
**General neuropsychology**					
Executive function					
WASI Matrices (/32)	26.81 (2.74)^j^	**11.80 (8.76)^a^**	22.40 (5.32)**	**19.10 (9.35)**	24.08 (6.73)**
Letter fluency (total)	15.93 (5.35)^d^	10.94 (5.86)^a^	8.88 (3.98)	9.00 (9.20)^i^	**7.42 (6.40)**
Category fluency (total)	24.07 (6.30)^d^	**11.41 (6.74)^a^**	**11.10 (6.36)**	**15.43 (11.59)^i^**	**6.67 (5.71)**
Working memory					
Digit span forward (max)	6.56 (1.03)^j^	5.79 (1.40)	4.38 (1.41)*,**	5.60 (1.27)*	6.67 (0.99)
Digit span reverse (max)	5.19 (1.17)^j^	3.21 (1.40)*	**3.62** (**1.30)**	**3.80 (1.93)**	4.92 (1.56)
Auditory input processing					
PALPA-3 (/36)	34.62 (1.66)^k^	NA	31.20 (5.78)^a^	31.40 (5.46)	33.67 (2.27)
Speech repetition					
Polysyllabic words (/45)	44.00 (1.55)^j^	NA	**41.30 (3.77)^h^**	**38.89 (8.02)^h^**	**40.67 (5.42)**
Short sentences (/10)	9.46 (0.88)^d^	NA	**5.29 (1.60)^h^**	**6.60 (2.80)**	**7.50 (2.11)**
Other language skills					
GNT (/30)	25.75 (2.46)^j^	**13.00 (7.22)**	**10.90 (6.94)**	**18.40 (8.82)**	**1.42 (4.32)**
BPVS (/150)	147.88 (2.09)^j^	135.32 (23.4)*	146.00 (3.25)*	127.50 (46.3)*	**73.50 (50.8)**
PALPA-55 (/24)	23.46 (1.20)^j^	NA	**19.00 (4.47)^h^**	**19.80 (5.12)**	**18.80 (6.09)**
Episodic memory					
RMT Faces (Short) (/25)	23.75 (2.5)^l^	**16.11 (3.26)^j^**	21.40 (3.65)^i^	22.83 (3.49)**^,f^	19.20 (3.69)^m^
RMT Faces (Long) (/50)	41.67 (3.70)^n^	**29.56 (5.57)^j^**	**29.00 (6.93)^e^**	35.50 (4.95)	**30.88 (3.36)^f^**
Other skills					
GDA calculation (/24)	14.81 (5.18)^j^	**5.06 (4.81)^i^**	**6.14 (4.41)^h^**	**7.00 (5.52)^h^**	10.18 (6.35)^h^
VOSP Object Decision(/20)	18.94 (1.48)^j^	**14.10 (3.60)**	**17.00 (1.41)^h^**	16.44 (4.59)**^,h^	**15.50 (3.78)^h^**

Mean (standard deviation) values and raw scores are presented (maximum value possible in parentheses), unless otherwise indicated; significant differences from healthy controls (*P* < 0.05) are in bold; *significantly different to svPPA (*P* < 0.05); **significantly different to AD (*P* < 0.05). Participants assessed in-person did the Mini-Mental State Examination (MMSE) while those assessed remotely did the T-MMSE. Similarly, the RMT Faces (Long) was administered to participants in-person, versus the RMT Faces (Short), which was administered to participants remotely (full details of our remote neuropsychological test battery are given in Heimbauer *et al*.^44^ A = ambidextrous; AD = patient group with typical Alzheimer’s disease; BPVS = British Picture Vocabulary Scale; Controls = healthy older control group; Digit span forward/reverse = maximum digit span recorded; GDA = Graded Difficulty Arithmetic; GNT = Graded Naming Test; L = left; lvPPA = patient group with logopenic variant primary progressive aphasia; NA = not available/applicable; nfvPPA = patient group with non-fluent/agrammatic variant primary progressive aphasia; PALPA = Psycholinguistic Assessments of Language Processing in Aphasia; R = right; RMT = Recognition Memory Test; svPPA = patient group with semantic variant primary progressive aphasia; Synonyms concrete/abstract = single-word comprehension of single words; T-MMSE = tele-MMSE; VOSP = Visual Object and Space Perception battery; WASI = Wechsler Abbreviated Scale of Intelligence.

Missing data for two participants.

See Supplementary material for details concerning the ‘best ear average’ measure.

Missing data for 15 participants.

Missing data for 10 participants.

Missing data for five participants.

Missing data for four participants.

Missing data for seven participants.

Missing data for one participant.

Missing data for three participants.

Missing data for nine participants.

Missing data for 12 participants.

Missing data for 21 participants.

Missing data for eight participants.

Missing data for 13 participants.

Owing to the Covid-19 pandemic, some data for this study were collected remotely (Supplementary material). We have described the design and implementation of our remote neuropsychological assessment protocol elsewhere.^52^

All participants gave informed consent to take part in the study. Ethical approval was granted by the UCL-NHNN Joint Research Ethics Committees, in accordance with Declaration of Helsinki guidelines.

### Creation of experimental stimuli

Lists of 50 different three-digit numbers (of the form, ‘five hundred and eighty-seven’; examples in Supplementary material) were recorded by two young adult female speakers in a Standard Southern British English accent with neutral prosody. They were recorded in Audacity (v 2.2.3), using a condenser microphone with a pop-shield in a sound-proof booth. Speech recordings were noise-vocoded using Matlab® (vR2019b) (https://uk.mathworks.com/) to generate acoustically altered stimuli with a prescribed level of degraded intelligibility (see Supplementary Fig. 1 for spectrograms). Details concerning the synthesis of noise-vocoded stimuli are provided in the Supplementary material. The vocoding intelligibility threshold for younger normal listeners is typically around three to four ‘channels’^36^; in this experiment, we noise-vocoded the speech recordings with 1 to 24 channels, sampling at each integer number of channels within this range to ensure we would be able to accurately capture even markedly abnormal psychometric functions in the patient cohort.

The final stimulus list comprised 100 different spoken three-digit numbers: four unvocoded (clear speech) and 96 noise-vocoded with four stimuli for each number of channels, ranging from 1 to 24.

### Experimental procedure

The stimuli were administered binaurally in a quiet room via Audio-Technica ATH-M50X headphones at a comfortable fixed listening level (at least 70 dB). Data for 30 participants were collected remotely via video link during the Covid-19 pandemic (Table 1 and Supplementary material).

To familiarize the participants with the experimental procedure, they were first asked to repeat five three-digit numbers (not included in the experimental session) that were spoken by the experimenter. Prior to presenting the experimental stimuli, participants were advised that the numbers they heard would vary in how difficult to understand they were, but that they should guess the number even if uncertain. Stimuli were presented in order of progressively decreasing channel number (intelligibility), first clear speech, then from 24 vocoding channels to one vocoding channel. On each experimental trial, the task was to repeat the number (or as many of the three digits as the participant could identify). Participants were allowed to write down the numbers they heard rather than speaking them if preferred; in scoring, we accepted the intended target digit as correct, even if imperfectly articulated. Responses were recorded for offline analysis. During the experiment, no feedback about performance was given and no time limits were imposed.

### Analysis of clinical and behavioural data

Data were analysed in MATLAB® (vR2019b) and in R® (v4). For continuous demographic and neuropsychological data, participant groups were compared using ANOVA and Kruskal-Wallis tests (dependent on normality of the data); group categorical data were compared using Fisher’s exact tests. Performance profiles in seven healthy control participants who performed the experiment both in person and subsequently remotely were very similar, justifying combining participants tested in person and remotely in the main analysis (Supplementary material). An alpha of 0.05 was adopted as the threshold for statistical significance on all tests.

Identification of noise-vocoded spoken numbers was scored according to the number of digits correct for each three-digit number (e.g. if the target number was ‘587’ and the participant responded ‘585’, they would score two points on that trial). As three digits were presented on every trial, this scoring effectively yielded a total of 12 (4 × 3) data-points for each vocoding channel number, for each participant. As the perceptual effect of noise-vocoding scales is exponential (e.g. the increase in intelligibility for normal listeners is much greater between two and four channels than between 20 and 24 channels), we applied a logarithmic (base 2) transformation to the data. The resulting data were then modelled using a Weibull sigmoid, a widely used function for fitting logarithmically scaled data.^53^ Individual participant and group mean psychometric curves were created for each diagnostic group using the MATLAB psignifit package. This package employs beta-binomial models that account for overdispersion of the fitted psychometric function, due (for example) to wide variation among individual patients.^53^ For each function, we report the following parameters: the binaural noise-vocoded speech intelligibility threshold (the number of vocoding channels at which 50% identification of noise-vocoded numbers was achieved); the slope of the function at the threshold point; lambda (the lapse rate, or number of incorrect responses at maximum performance asymptote); gamma (the guess rate, or number of correct responses at minimum performance level); and eta (a measure of overdispersion).

As the data were not normally distributed, we used non-parametric Kruskal Wallis tests to analyse psychometric parameters. Where the omnibus test was significant, we conducted Dunn’s tests to conduct pairwise comparisons between participant groups. We assessed the relationship of noise-vocoded speech intelligibility threshold to forward digit span over the whole patient cohort, using Spearman’s correlation; here, digit span provides a metric of each patient’s overall ability to repeat (hear, hold in short term memory and articulate) natural spoken numbers. We further used Spearman’s correlation to assess, over the combined patient cohort, the relationship of intelligibility threshold to general demographic (age, sex) and clinical [symptom duration, Mini-Mental State Examination (MMSE) score] variables, executive performance [Wechsler Abbreviated Scale of Intelligence (WASI) Matrices] and measures of auditory perceptual function (pure tone audiometry, phonemic pairs discrimination on the Psycholinguistic Assessment of Language Processing in Aphasia (PALPA)-3 subtest) (Supplementary material).

Finally, receiver operating characteristic (ROC) curves were derived to assess the overall diagnostic utility of noise-vocoded speech comprehension in distinguishing each patient group from healthy controls. The binary classifier used was the 50% speech intelligibility threshold obtained from each psychometric function. The area under the ROC curve (AUC) was calculated for each syndromic group using parametric estimates in the pROC R package.^54,55^

### Brain image acquisition and analysis

Volumetric brain magnetic resonance images were acquired for 25 patients in a 3 T Siemens Prisma MRI scanner, using a 32-channel phased array head coil and following a T_1_-weighted sagittal 3D magnetization prepared rapid gradient echo (MPRAGE) sequence (echo time = 2.9 ms, inversion time = 900 ms, repetition time = 2200 ms), with dimensions 256 mm × 256 mm × 208 mm and voxel size 1.1 mm × 1.1 mm × 1.1 mm.

For the VBM analysis, patients’ brain images were first preprocessed and normalized to MNI space using SPM12 software (http://www.fil.ion.ucl.ac.uk/spm/software/spm12/) and the DARTEL toolbox with default parameters running under MATLAB R2014b. Images were smoothed using a 6-mm full-width at half-maximum (FWHM) Gaussian kernel. To control for individual differences in total (pre-morbid) brain size, total intracranial volume was calculated for each participant by summing white matter, grey matter and CSF volumes post-segmentation.^56^ An explicit brain mask was created using an automatic mask-creation strategy designed previously.^57^ A study-specific mean brain template image upon which to overlay statistical parametric maps was created by warping all patients’ native-space whole-brain images to the final DARTEL template and using the ImCalc function to generate an average of these images.

We assessed grey matter associations of noise-vocoded speech intelligibility threshold over the combined patient cohort. Voxel-wise grey matter intensity was modelled as a function of performance threshold in a multiple regression design, incorporating age, total intracranial volume and diagnostic group membership as covariates. Statistical parametric maps were generated using an initial cluster-defining threshold (*P* < 0.001) and assessed at peak-level significance threshold *P* < 0.05, after family-wise error (FWE) correction for multiple voxel-wise comparisons within five separate predefined regions of interest, specified during the design of the study, and based on previously published work on degraded speech perception in the healthy brain and in neurodegenerative disease: these regions, which together constitute a distributed neural network processing degraded speech signals, comprised left planum temporale,^38,39^ left angular gyrus,^40–42^ left anterior superior temporal gyrus,^40,58,59^ left inferior frontal gyrus ^40,48,58^ and left cingulate gyrus.^40,60^ Anatomical volumes were derived from Oxford-Harvard cortical maps^61^ and are shown in Supplementary Fig. 3.

### Data availability

The data that support the findings of this study are available on request from the corresponding author. The data are not publicly available because they contain information that could compromise the privacy of research participants.

## Results

### General participant group characteristics

Participant groups did not differ significantly in sex distribution, handedness or years of formal education (all *P* > 0.05, Table 1). Patient groups differed significantly in terms of age (*P* = 0.04), with the Alzheimer’s disease (*z* = 2.22, *P* = 0.03), lvPPA (*z* = 2.47, *P* = 0.01) and nfvPPA (*z* = 2.75, *P* = 0.01) PPA groups being older on average than the svPPA group. Patient groups did not differ in mean symptom duration (*P* = 0.09) but did differ in MMSE score [*H*(3) = 11.3, *P* = 0.01; Table 1], the Alzheimer’s disease group performing worse than the nfvPPA (*z* = −3.22, *P* = 0.001) and svPPA (*z* = −2.10, *P* = 0.04) groups. General neuropsychological profiles were in keeping with syndromic diagnosis for each patient group (Table 1). Pure tone audiometry (in the participant subcohort assessed in-person) revealed no substantial peripheral hearing deficits nor any significant differences between participant groups. Basic speech discrimination (assessed using the PALPA-3) did not differ significantly from the healthy control group for any of the PPA syndromic groups.

### Experimental behavioural data

**Figure 1:**
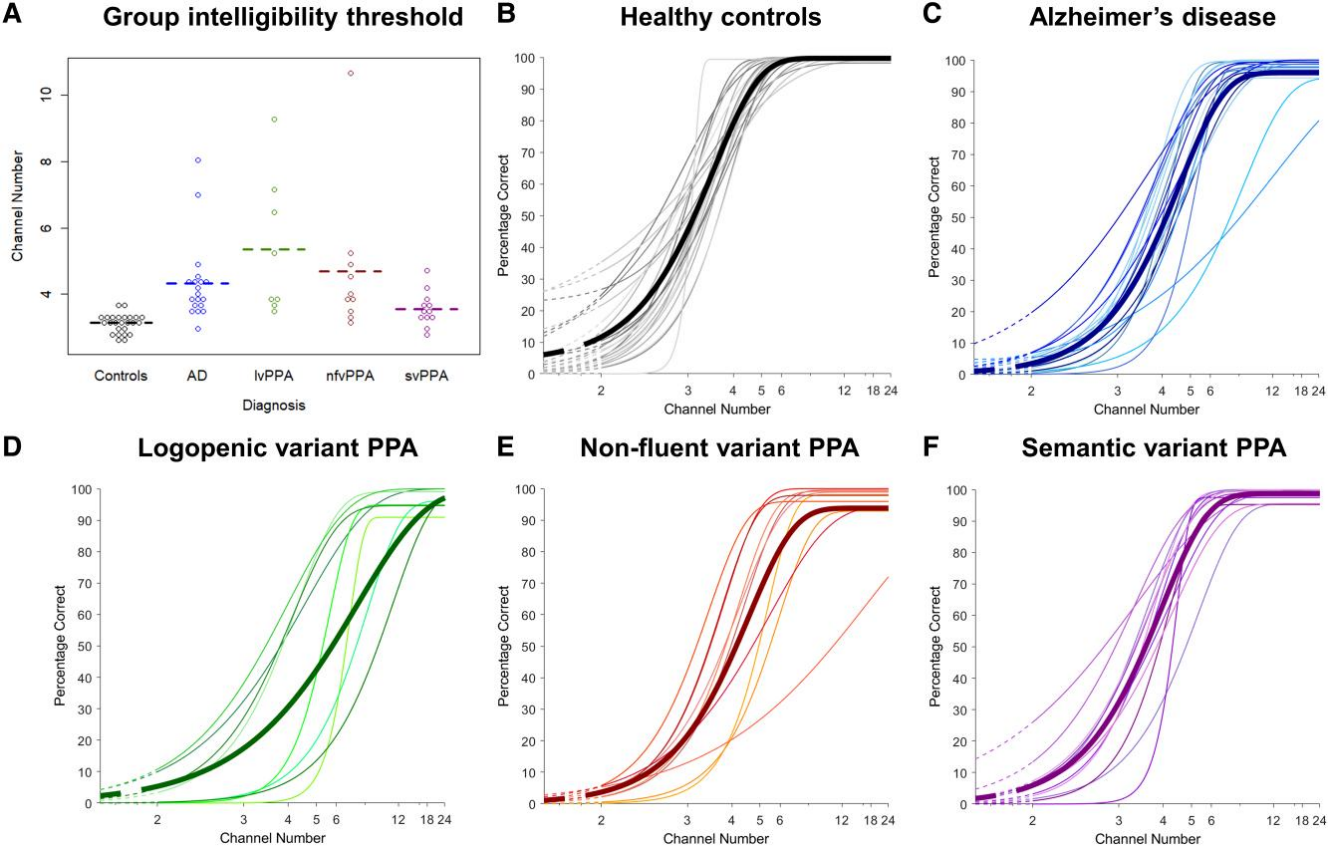
**Beeswarm plots of individual participants’ speech intelligibility threshold and psychometric curves for comprehension of noise-vocoded speech within each diagnostic group**. (**A**) Group speech intelligibility threshold values correspond to number of vocoding channels in the speech stimulus at which 50% intelligibility of spoken numbers was achieved. Dashed lines represent the mean for each group. (**B**–**F**) The *y*-axis here shows the percentage of digits identified correctly (from a total of 12 digits) at each noise-vocoding level; the *x*-axis shows the number of vocoding channels, plotted on a log scale. (**B**) Combined psychometric curves of all healthy control participants, with the bolded line indicating mean [curves have been fitted through values (coloured dots) representing the mean score correct across individual participants in that group at each noise-vocoding level]. (**C**) Combined psychometric curves of all the participants with Alzheimer’s disease (AD), with the bold line indicating mean (as in **B**). (**D**) Combined psychometric curves of all the participants with logopenic variant primary progressive aphasia (lvPPA), with the bold line indicating mean (as in **B**). (**E**) Combined psychometric curves of all the participants with non-fluent variant primary progressive aphasia (nfvPPA), with the bold line indicating mean (as in **B**). (**F**) Combined psychometric curves of all the participants with semantic variant primary progressive aphasia (svPPA), with the bold line indicating mean (as in **B**).

**Figure 2:**
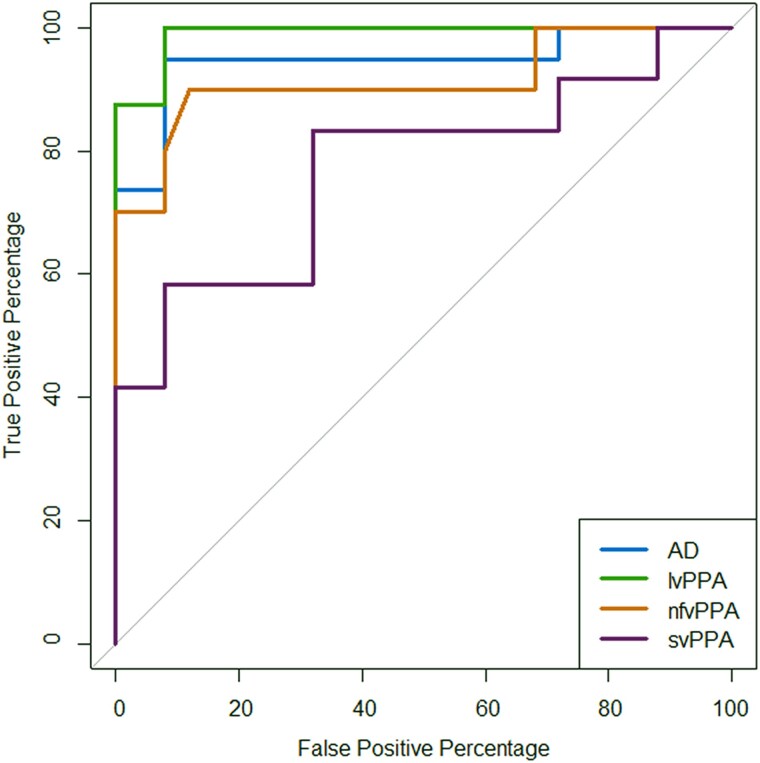
**ROC curves for comprehension of noise-vocoded speech in patient groups versus healthy older controls.** Receiver operating characteristic (ROC) curves for each syndromic group versus the healthy older control group are shown; the binary classifier used was the speech intelligibility threshold obtained in the psychometric functions (Table 2). An area under the curve (AUC) of 1 would correspond to an ideal classifier. AUC values obtained were as follows: Alzheimer’s disease (AD), AUC = 0.95, 95% CI (0.87, 1); logopenic variant primary progressive aphasia (lvPPA), AUC = 0.99, 95% CI (0.97, 1); non-fluent/agrammatic variant primary progressive aphasia (nfvPPA) AUC = 0.91, 95% CI (0.78, 1); semantic variant primary progressive aphasia (svPPA), AUC = 0.77, 95% CI (0.59, 0.96). CI = confidence interval.

Psychometric parameters for the participant groups are presented in Table 2. Individual and mean psychometric functions and data-points of the noise-vocoded speech intelligibility threshold are presented in Fig. 1. Additional data-point plots of the slope at 50% correct and lapse rate are presented in Supplementary Fig. 2. ROC curves for the patient groups versus the healthy control group are shown in Fig. 2. Exclusion of two upper bound outliers on speech intelligibility threshold (>97.5 quantile) in parallel analyses left the results qualitatively unaltered. Results from the full dataset are accordingly reported in-text below; parallel analyses with outliers removed are reported in the Supplementary material.

**Table 2:** Psychometric function parameters for comprehension of noise-vocoded speech in each participant group

Parameter	Controls		AD		lvPPA		nfvPPA		svPPA		Effect size
	Mean (SD)	95% CI	Mean (SD)	95% CI	Mean (SD)	95% CI	Mean (SD)	95% CI	Mean (SD)	95% CI	η^2^*_H_*
Threshold	3.14 (0.27)	3.03, 3.25	**4.33 (1.25)**	3.73, 4.93	**5.35 (2.13)**	3.57, 7.13	**4.68 (2.22)**	3.09, 6.27	3.55* (0.53)	3.21, 3.89	0.500
Slope	1.08 (0.90)	0.71, 1.45	0.79 (0.27)	0.66, 0.92	0.82 (0.36)	0.52, 1.12	0.81 (0.27)	0.62, 1.00	0.95 (0.46)	0.66, 1.24	0.007
Lambda (lapse rate)	0.00 (0.01)	0.00, 0.00	**0.02 (0.02)**	0.01, 0.03	**(0.03, 0.03)**	0.01, 0.06	**0.02 (0.02)**	0.01, 0.03	**0.02 (0.03)**	0.00, 0.04	0.174
Gamma (guess rate)	0.04 (0.07)	0.01, 0.07	**0.00 (0.01)**	−0.00, 0.01	**(0.00, 0.00)**	0.00, 0.00	**0.00 (0.00)**	0.00, 0.00	**0.00 (0.00)**	0.00, 0.00	0.181
Eta	0.00 (0.00)	0.00, 0.00	0.04 (0.10)	0.04, 0.05	0.04 (0.10)	−0.04, 0.12	0.00 (0.00)	0.00, 0.00	0.00 (0.00)	0.00, 0.00	0.049

Parameters are based on mean psychometric functions for each participant group (see text and Supplementary Fig. 2); mean (standard deviation, SD) and confidence intervals (CI) for each parameter values are shown. Threshold indicates 50% intelligibility of noise-vocoded spoken numbers; slope indicates the slope of the psychometric function at this threshold point; lambda (lapse rate) indicates the number of incorrect responses at the maximum performance level; gamma (guess rate) indicates the number of correct responses at the minimum performance level; eta (overdispersion) indicates scaling of extra variance (a value near 0 indicates that the data are essentially binomially distributed, while values near 1 indicate severely overdispersed data). The η^2^*_H_* (eta-squared) parameter measures effect size of the omnibus test for each parameter, and is expressed as a proportion ranging from 0 to 1, with higher values representing larger effect sizes. Significant differences (*P* < 0.05) between patient groups and the healthy older control group are shown in bold; *significantly lower in the svPPA group than the other patient groups (all *P* < 0.05). AD = patient group with typical Alzheimer’s disease; Controls = healthy older control group; lvPPA = patient group with logopenic variant primary progressive aphasia; nfvPPA = patient group with non-fluent/agrammatic variant primary progressive aphasia; svPPA = patient group with semantic variant primary progressive aphasia.

There was a significant main effect of diagnostic group on noise-vocoded speech intelligibility threshold [*H*(4) = 38.48, *P* < 0.001]. In *post hoc* pairwise group comparisons versus healthy controls, mean intelligibility threshold was significantly elevated in all patient groups: in the lvPPA (*z* = 4.48, *P* < 0.001), nfvPPA (*z* = 3.97, *P* < 0.001), Alzheimer’s disease (*z* = 5.08, *P* < 0.001) and svPPA (*z* = 2.23, *P* = 0.03) groups. Comparing patient groups, intelligibility threshold was significantly elevated in the Alzheimer’s disease (*z* = 2.07, *P* = 0.04) and lvPPA (*z* = 2.27, *P* = 0.02) groups compared with the svPPA group. There was no significant effect of diagnostic group on the slope of the psychometric function (*P* = 0.347). There was a significant main effect of diagnostic group on the lapse rate, lambda [*H*(4) = 16.03, *P* = 0.003]. In *post hoc* pairwise group comparisons versus healthy controls, there was a significantly higher lapse rate (more errors made at maximum performance) in all patient groups: in the lvPPA (*z* = 2.68, *P* = 0.007), Alzheimer’s disease (*z* = 2.61, *P* = 0.009), nfvPPA (*z* = 3.27, *P* = 0.001), and svPPA (*z* = 2.31, *P* = 0.02) groups. There were no significant differences between patient groups for lapse rate. There was a significant main effect of diagnostic group on the guess rate, gamma [*H*(4) = 16.49, *P* = 0.002]. In *post hoc* pairwise group comparisons, there was a significantly higher gamma rate (i.e. more correct answers made at minimum performance) in the healthy control group than any patient groups (*P* < 0.05). There was no significant effect of diagnostic group on eta (overdispersion of the data) of the psychometric function (*P* = 0.118). Group effect sizes (Table 2) were large for intelligibility threshold, lapse rate and gamma rate, but small for other psychometric parameters.^62,63^

Individual variability in psychometric parameters within participant groups was substantial (Fig. 1 and Table 2). Most pertinently, variation in noise-vocoded speech intelligibility threshold was wider in the Alzheimer’s disease group than in healthy controls and most marked in the lvPPA and nfvPPA groups.

Over the combined patient cohort, noise-vocoded speech intelligibility threshold was not significantly correlated with peripheral hearing function (*r* = −0.04, *P* = 0.856), phonological discrimination in clear speech (PALPA-3 score; *r* = −0.25, *P* = 0.185), age (*r* = 0.21, *P* = 0.152) or symptom duration (*r* = −0.04, *P* = 0.775). Intelligibility threshold in the patient cohort was significantly correlated with WASI Matrices score (*r* = −0.46, *P* = 0.001), MMSE score (*r* = −0.53, *P* < 0.001) and forward digit span (*r* = −0.63, *P* < 0.001) (Supplementary Fig. 4). Lapse rate was also significantly correlated with forward digit span across the combined patient cohort (*r* = −0.34, *P* = 0.018) (Supplementary Fig. 4).

Analysis of ROC curves revealed that noise-vocoded speech intelligibility threshold discriminated all patient groups well from healthy controls. Based on AUC values (where a value of 1 would indicate an ideal classifier and values >0.8 a clinically robust discriminator^64,65^), discrimination was ‘excellent’ for the lvPPA group [AUC 0.99, 95% confidence interval (CI) (0.97, 1)], Alzheimer’s disease group [AUC 0.95, 95% CI (0.87, 1)] and nfvPPA group [AUC 0.91, 95% CI (0.78, 1)] and ‘fair’ for the svPPA group [AUC 0.77, 95% CI (0.59, 0.96)].

### Neuroanatomical data

**Figure 3:**
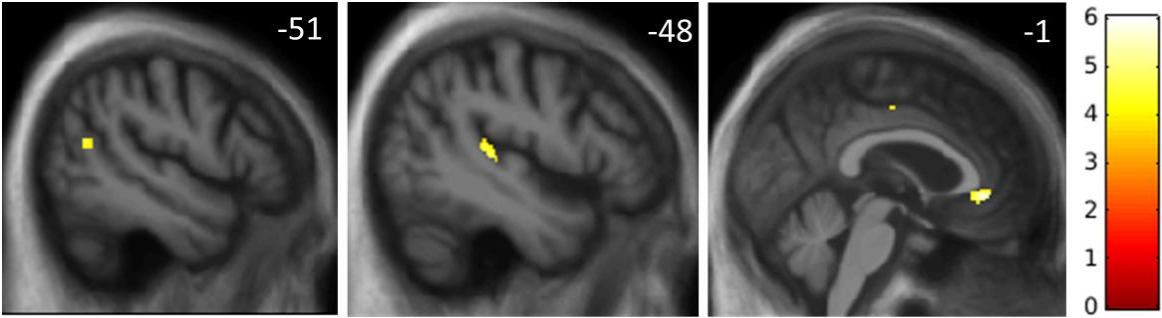
**Statistical parametric maps of regional grey matter atrophy associated with elevated noise-vocoded speech intelligibility threshold in the combined patient cohort**. Maps are rendered on sagittal sections of the group mean T_1_-weighted magnetic resonance image in MRI space, thresholded at *P* < 0.001 uncorrected for multiple voxel-wise comparisons, and masked using the pre-specified neuroanatomical region of interests (as used in the small volume corrections) that were significant at *P* < 0.05_FWE_ for multiple comparisons, over the whole brain for display purposes. The colour bar (*right*) codes voxel-wise *t*-values. All sections are through the left cerebral hemisphere; the plane of each section is indicated using the corresponding MNI coordinate (mm).

Statistical parametric maps of grey matter regions associated with speech intelligibility threshold are shown in Fig. 3 and local maxima are summarized in Table 3. Correlation plots for each significant peak voxel with speech intelligibility threshold are shown in Supplementary Fig. 5.

**Table 3:** Neuroanatomical associations of noise-vocoded speech intelligibility threshold in the patient cohort

Region	Cluster size (voxels)	Peak (mm)			T score	*P*_FWE_
		*x*	*y*	*z*		
Left planum temporale	131	−48	−31	6	4.65	0.019
Left angular gyrus	36	−51	−61	16	4.51	0.037
Left cingulate gyrus	142	−1	38	−5	5.68	0.012

The table shows significant negative associations between regional grey matter volume and intelligibility threshold for noise-vocoded speech, based on the voxel-based morphometric analysis of brain magnetic resonance images for the combined patient cohort. Coordinates of peaks (local maxima) are in MNI standard space. Local maxima shown were significant (*P* < 0.05) after family-wise error (FWE) correction for multiple voxel-wise comparisons within the pre-specified anatomical regions of interest (see text and Supplementary Fig. 2).

Across the combined patient cohort, intelligibility threshold was significantly negatively associated with regional grey matter volume (i.e. associated with grey matter atrophy) in left planum temporale, left angular gyrus and anterior cingulate gyrus (all *P*_FWE_ < 0.05 after correction for multiple voxel-wise comparisons within the relevant pre-specified neuroanatomical region of interest).

## Discussion

Here we have shown that perception of acoustically degraded (noise-vocoded) speech is impaired in patients with Alzheimer’s disease and PPA syndromes relative to healthy older listeners, and further, stratifies syndromes: impairment was most severe in lvPPA and nfvPPA, and significantly more severe in Alzheimer’s disease than in svPPA. Intelligibility threshold for noise-vocoded speech did not correlate with measures of pure tone detection or phoneme discrimination in clear speech, suggesting that the deficit does not simply reflect a problem with peripheral hearing or elementary speech perception. Individual noise-vocoded speech intelligibility threshold varied widely within the Alzheimer’s disease, lvPPA and nfvPPA groups. Our findings suggest that elevation in noise-vocoded speech intelligibility threshold in these dementia syndromes captures a central auditory impairment potentially relevant to difficulties in diverse everyday listening situations requiring the decoding of acoustically altered speech signals.

Neuroanatomically, impaired noise-vocoded speech comprehension across dementia syndromes was underpinned by atrophy of left planum temporale, angular gyrus and anterior cingulate gyrus. This cortical network has been shown to be critical for processing speech signals under a range of noisy, daily listening conditions.^5,32,33,42,66^ Planum temporale is likely to play a fundamental role in the deconvolution of complex sound patterns and engagement of neural representations corresponding to phonemes and other auditory objects.^38,39,67^ Angular gyrus mediates the disambiguation of speech signals in challenging listening environments, working memory for speech signals and transcoding of auditory inputs for motor responses, including orienting and repetition.^41,67–70^ Both regions are targeted in Alzheimer’s disease, lvPPA and nfvPPA^71–74^ and have been particularly implicated in the pathogenesis of impaired speech perception in these diseases.^29,30,32,75^ The anterior cingulate cortex operates in concert with these more posterior cortical hubs to decode spoken messages under challenging listening conditions,^40,60^ with a more general role in cognitive control and in allocating attentional resources to salient stimuli.^66,76,77^ Reduced activation of the anterior cingulate cortex during tracking of information in degraded speech signals has been demonstrated in nfvPPA and svPPA.^33^

These neuroanatomical considerations suggest that the mechanisms of impaired noise-vocoded speech intelligibility are likely to differ between neurodegenerative syndromes, in keeping with the dissociable processes involved in phoneme recognition.^2^ Noise-vocoding fundamentally reduces the availability of acoustic cues that define phonemes as auditory objects: impaired recognition of these degraded auditory objects could in principle result from deficient encoding of acoustic features, damaged object–level representations (the auditory analogue of ‘apperceptive’ deficits in the visual domain) or impaired top-down, predictive disambiguation based on stored knowledge about speech signal characteristics. In Alzheimer’s disease and lvPPA, a core deficit of object-level representations has been demonstrated neuropsychologically and electrophysiologically using other procedures that alter acoustic detail in phonemes and non-verbal sounds^31,33,78,79^; it is therefore plausible that an analogous apperceptive deficit may have impacted the recognition of noise-vocoded phonemes in the Alzheimer’s disease and lvPPA groups here. In nfvPPA, one previous MEG study of noise-vocoded speech perception has foregrounded the role of inflexible top-down predictive decoding mechanisms (i.e. inappropriately ‘precise’ stored expectations about incoming speech signals, leading to delayed disambiguation of degraded speech), instantiated in frontal cortex.^48^ However, this is a clinically, neuroanatomically and neuropathologically diverse syndrome, and involvement of posterior superior temporal cortex engaged in early auditory pattern analysis may constitute a ‘second hit’ to phoneme recognition.^33,78,80,81^ In svPPA, the elevated noise-vocoded intelligibility threshold is *a priori* more likely to reflect reduced activation of semantic mechanisms engaged in the predictive disambiguation of degraded speech signals; and indeed, comprehension of other kinds of acoustically degraded speech signals by patients with svPPA has previously been shown to be sensitive to semantic predictability and to engage anterior cingulate cortex.^29,31,33^

Increasing intelligibility threshold was correlated with digit span over the combined patient cohort. This suggests that verbal working memory limitations may be integrally related to impaired processing of degraded speech, consistent with previous work highlighting the role of working memory in speech perception, particularly in older adults.^82,83^ As working memory demands did not vary across trials and number of vocoding channels, the principal driver of intelligibility threshold is likely to have been the level of acoustic alteration in the speech signal. On the other hand, all patient groups showed an increased lapse rate (i.e. errors unrelated to the stimulus level^53^) at higher vocoding channel numbers (i.e. for minimally noise-vocoded speech signals approaching clear speech). This echoes previous work demonstrating that active listening can be abnormal in lvPPA and nfvPPA even for clear speech and other sounds in quiet.^75,84^ As lapse rate was also correlated with digit span, this suggests that reduced working memory may influence performance at the upper asymptote, potentially interacting with top-down mechanisms engaged in the predictive processing of speech.^48^ Indeed, frontal processes are likely to play a broader role in the disambiguation of degraded speech signals, including the allocation of attentional and executive resources^85^ and according with the observed correlation here between noise-vocoded speech intelligibility threshold and WASI Matrices score. Taken together, the present findings corroborate the profiles of deficit previously documented in Alzheimer’s disease and PPA syndromes for comprehension of sinewave speech and phonemic restoration in noise-interrupted speech.^29,31^

Our findings further suggest that markers of noise-vocoded speech comprehension may have diagnostic and biomarker potential. The ROC analysis on the noise-vocoded intelligibility threshold measure (Fig. 2) suggests that it would constitute an ‘excellent’ clinical test (corresponding to AUC > 0.9) for discriminating patients with Alzheimer’s disease, lvPPA and nfvPPA from healthy older individuals.^65^ However, the smaller sample size does need to be taken into consideration for the ROC analysis. Additionally, the noise-vocoded intelligibility threshold was correlated with overall disease severity (MMSE score) in the patient cohort. These findings build on a growing body of work suggesting that markers of ‘central’ hearing (auditory cognition) may sensitively signal the functional integrity of cortical regions that are vulnerable to Alzheimer’s disease and other neurodegenerative pathologies.^5,8,16^ The results of this study could further motivate the development of tailored strategies to help manage hearing difficulties experienced by people with dementia in various daily-life contexts and environments.

This study has limitations that suggest directions for further work. Our noise-vocoding paradigm (based on a step-wise linear progression through channel numbers) was not optimally efficient; an adaptive staircase procedure would reduce testing time and allow individual thresholds to be captured without administering uninformative trials at higher channel numbers. It would be relevant to assess to what extent patients’ comprehension of noise-vocoded speech can be modulated: pharmacologically (in particular, by acetylcholinesterase inhibitors^30^) and/or by perceptual learning, as in healthy listeners.^86–88^ Using another kind of speech degradation (sinewave transformation), we have previously shown that pharmacological and perceptual learning effects may operate in Alzheimer’s disease and PPA syndromes.^29,30^ To establish how noise-vocoded speech perception and its modulatory factors relate to neural circuit integrity in Alzheimer’s disease PPA, functional neuroimaging using techniques such as functional MRI and MEG will be required to capture dynamic network connectivity engaged by these processes and the neural mechanisms that represent and decode vocoded speech sounds. Furthermore, whilst a direct comparison across sensory modalities was beyond the scope of the present study, the perceptual processing deficit presented here in the auditory domain may extend to other sensory domains, such as vision.^89^ It would be of particular interest to assess whether cross-modal sensory cues can be used to help disambiguate degraded speech signals in patients with Alzheimer’s disease and PPA.

From a clinical perspective, this work should be taken forward in several ways. The group sizes here were relatively small: the noise-vocoding paradigm should be extended to larger patient cohorts, which (given the comparative rarity of PPA) will likely entail multicentre collaboration. Besides corroborating the present group findings, assessment of larger cohorts would allow characterization of the sources of the wide individual variation within diagnostic groups. There is also a need for prospective, longitudinal studies—both to assess how markers of degraded speech perception relate to disease course and to determine how early such markers may signal underlying neurodegenerative pathology. Auditory measures based on degraded speech comprehension would be well suited to future digital applications and potentially to large-scale screening of populations at risk of incident Alzheimer’s disease, as well as outcome measures in clinical trials of pharmacotherapies and non-pharmacological interventions.^8,16^ Our work adds to a growing body of evidence that central hearing problems may emerge as early and/or prominent symptoms in dementia syndromes.^8^ Improved awareness and understanding of these issues in healthcare professionals such as audiologists and neurologists could inform care, management and counselling of patients. Older hearing aid users at risk of dementia are likely to be particularly vulnerable to impaired central mechanisms of degraded speech comprehension, given that the quality of incoming acoustic information in this setting is already compromised.

The key next step, however, will be to establish how well measures of degraded speech comprehension, not solely noise-vocoding but also other ethologically relevant adverse speech listening tests, correlate with daily-life hearing and communication in Alzheimer’s disease and other neurodegenerative diseases—using both currently standardized symptom questionnaires and bespoke instruments developed to capture functional hearing disability in dementia. We have previously shown that pure tone audiometry alone is a poor predictor of everyday hearing^90^ while degraded speech performance may have better predictive value in patients with dementia.^91^ There would be considerable clinical value in a quantifiable index of degraded speech perception that could serve as a proxy and predictor of daily life hearing function and disability in major dementias: comprehension of noise-vocoded speech is a promising candidate.

The link between hearing impairment and dementia continues to be debated but presents a major opportunity for earlier diagnosis and intervention. Our findings suggest that the perception of degraded (noise-vocoded) speech quantifies central hearing functions beyond sound detection in dementia and stratifies major dementia syndromes. This central hearing index may constitute a proxy for the communication difficulties experienced by patients with Alzheimer’s disease and PPA under challenging listening conditions in daily life. We hope that this work will motivate further studies to define the diagnostic and therapeutic scope of central hearing measures based on degraded speech perception in these diseases.

## Supplementary Material

awad163_Supplementary_Data

## References

[1] Bregman AS . Auditory scene analysis: The perceptual organization of sound. MIT Press; 1994.

[2] Goll JC , Crutch SJ, Warren JD. Central auditory disorders: Toward a neuropsychology of auditory objects. Curr Opin Neurol. 2010;23:617. 20975559 10.1097/WCO.0b013e32834027f6 PMC3374998

[3] Griffiths TD , Warren JD. What is an auditory object? Nature Reviews Neuroscience. 2004;5:887. 15496866 10.1038/nrn1538

[4] Lin FR , Metter EJ, O’Brien RJ, Resnick SM, Zonderman AB, Ferrucci L. Hearing loss and incident dementia. Arch Neurol. 2011;68:214–220. 21320988 10.1001/archneurol.2010.362 PMC3277836

[5] Jiang J , Benhamou E, Waters S, et al Processing of degraded speech in brain disorders. Brain Sci. 2021;11:394. 33804653 10.3390/brainsci11030394 PMC8003678

[6] Gates GA , Anderson ML, Feeney MP, McCurry SM, Larson EB. Central auditory dysfunction in older persons with memory impairment or Alzheimer dementia. Archives of Otolaryngology–Head & Neck Surgery. 2008;134:771. 18645130 10.1001/archotol.134.7.771 PMC2871110

[7] Gates GA , Anderson ML, McCurry SM, Feeney MP, Larson EB. Central auditory dysfunction as a harbinger of Alzheimer dementia. Arch Otolaryngol Head Neck Surg. 2011;137:390–395. 21502479 10.1001/archoto.2011.28 PMC3170925

[8] Johnson JCS , Marshall CR, Weil RS, Bamiou D-E, Hardy CJD, Warren JD. Hearing and dementia: From ears to brain. Brain. 2021;144:391–401. 33351095 10.1093/brain/awaa429 PMC7940169

[9] Livingston G , Sommerlad A, Orgeta V, et al Dementia prevention, intervention, and care. Lancet. 2017;390:2673–2734. 28735855 10.1016/S0140-6736(17)31363-6

[10] Griffiths TD , Lad M, Kumar S, et al How can hearing loss cause dementia? Neuron. 2020;108:401–412. 32871106 10.1016/j.neuron.2020.08.003 PMC7664986

[11] Powell DS , Oh ES, Reed NS, Lin FR, Deal JA. Hearing loss and cognition: What we know and where we need to go. Front Aging Neurosci. 2022;13:769405. 35295208 10.3389/fnagi.2021.769405 PMC8920093

[12] Loughrey DG , Kelly ME, Kelley GA, Brennan S, Lawlor BA. Association of age-related hearing loss with cognitive function, cognitive impairment, and dementia: A systematic review and meta-analysis. JAMA Otolaryngology–Head & Neck Surgery. 2018;144:115–126. 29222544 10.1001/jamaoto.2017.2513 PMC5824986

[13] Tarawneh HY , Menegola HK, Peou A, Tarawneh H, Jayakody DMP. Central auditory functions of Alzheimer’s disease and its preclinical stages: A systematic review and meta-analysis. Cells. 2022;11:1007. 35326458 10.3390/cells11061007 PMC8947537

[14] Tarawneh HY , Mulders WHAM, Sohrabi HR, Martins RN, Jayakody DMP. Investigating auditory electrophysiological measures of participants with mild cognitive impairment and Alzheimer’s disease: A systematic review and meta-analysis of event-related potential studies. Alzheimers Dement. 2021;17(S5):e058497. 10.3233/JAD-210556 PMC8609695 34569950

[15] Pronk M , Lissenberg-Witte BI, van der Aa HPA, et al Longitudinal relationships between decline in speech-in-noise recognition ability and cognitive functioning: The longitudinal aging study Amsterdam. J Speech Lang Hear Res. 2019;62(4s):1167–1187. 31026198 10.1044/2018_JSLHR-H-ASCC7-18-0120

[16] Stevenson JS , Clifton L, Kuźma E, Littlejohns TJ. Speech-in-noise hearing impairment is associated with an increased risk of incident dementia in 82,039 UK biobank participants. Alzheimers Dement. 2022;18:445–456. 34288382 10.1002/alz.12416

[17] Golden HL , Agustus JL, Goll JC, et al Functional neuroanatomy of auditory scene analysis in Alzheimer's disease. Neuroimage Clin. 2015;7:699–708. 26029629 10.1016/j.nicl.2015.02.019 PMC4446369

[18] Bouma A , Gootjes L. Effects of attention on dichotic listening in elderly and patients with dementia of the Alzheimer type. Brain Cogn. 2011;76:286–293. 21429649 10.1016/j.bandc.2011.02.008

[19] Idrizbegovic E , Hederstierna C, Dahlquist M, Kämpfe Nordström C, Jelic V, Rosenhall U. Central auditory function in early Alzheimer's disease and in mild cognitive impairment. Age Ageing. 2011;40:249–254. 21233090 10.1093/ageing/afq168

[20] Utoomprurkporn N , Hardy CJD, Stott J, Costafreda SG, Warren J, Bamiou DE. “The dichotic digit test” as an Index indicator for hearing problem in dementia: Systematic review and meta-analysis. J Am Acad Audiol. 2020;31:646–655. 33296935 10.1055/s-0040-1718700

[21] Goll JC , Kim LG, Ridgway GR, et al Impairments of auditory scene analysis in Alzheimer's disease. Brain. 2012;135(Pt 1):190–200. 22036957 10.1093/brain/awr260 PMC3267978

[22] Golden HL , Agustus JL, Nicholas JM, et al Functional neuroanatomy of spatial sound processing in Alzheimer's disease. Neurobiol Aging. 2016;39:154–164. 26923412 10.1016/j.neurobiolaging.2015.12.006 PMC4782736

[23] Golden HL , Nicholas JM, Yong KXX, et al Auditory spatial processing in Alzheimer’s disease. Brain. 2015;138:189–202. 25468732 10.1093/brain/awu337 PMC4285196

[24] Hardy CJD , Yong KXX, Goll JC, Crutch SJ, Warren JD. Impairments of auditory scene analysis in posterior cortical atrophy. Brain. 2020;143:2689–2695. 32875326 10.1093/brain/awaa221 PMC7523698

[25] Bidelman GM , Howell M. Functional changes in inter- and intra-hemispheric cortical processing underlying degraded speech perception. Neuroimage. 2016;124(Pt A):581–590. 26386346 10.1016/j.neuroimage.2015.09.020

[26] Burda AN , Hageman CF, Brousard KT, Miller AL. Dementia and identification of words and sentences produced by native and nonnative English speakers. Percept Mot Skills. 2004;98(3 Pt 2):1359–1362. 15291227 10.2466/pms.98.3c.1359-1362

[27] Hailstone JC , Ridgway GR, Bartlett JW, Goll JC, Crutch SJ, Warren JD. Accent processing in dementia. Neuropsychologia. 2012;50:2233–2244. 22664324 10.1016/j.neuropsychologia.2012.05.027 PMC3484399

[28] Fletcher PD , Downey LE, Agustus JL, et al Agnosia for accents in primary progressive aphasia. Neuropsychologia. 2013;51:1709–1715. 23721780 10.1016/j.neuropsychologia.2013.05.013 PMC3724054

[29] Hardy CJD , Marshall CR, Bond RL, et al Retained capacity for perceptual learning of degraded speech in primary progressive aphasia and Alzheimer's disease. Alzheimers Res Ther. 2018;10:70. 30045755 10.1186/s13195-018-0399-2 PMC6060531

[30] Hardy CJD , Hwang YT, Bond RL, et al Donepezil enhances understanding of degraded speech in Alzheimer's disease. Ann Clin Transl Neurol. 2017;4:835–840. 29159197 10.1002/acn3.471 PMC5682113

[31] Jiang J , Johnson JCS, Requena-Komuro M-C, et al Phonemic restoration in Alzheimer’s disease and semantic dementia: A preliminary investigation. Brain Commun. 2022;4:fcac118. 35611314 10.1093/braincomms/fcac118 PMC9123842

[32] Hardy CJD , Agustus JL, Marshall CR, et al Behavioural and neuroanatomical correlates of auditory speech analysis in primary progressive aphasias. Alzheimers Res Ther. 2017;9:53. 28750682 10.1186/s13195-017-0278-2 PMC5531024

[33] Hardy CJD , Agustus JL, Marshall CR, et al Functional neuroanatomy of speech signal decoding in primary progressive aphasias. Neurobiol Aging. 2017;56:190–201. 28571652 10.1016/j.neurobiolaging.2017.04.026 PMC5476347

[34] Gates GA , Mills JH. Presbycusis. Lancet. 2005;366:1111–1120. 16182900 10.1016/S0140-6736(05)67423-5

[35] Holmes E , Zeidman P, Friston KJ, Griffiths TD. Difficulties with speech-in-noise perception related to fundamental grouping processes in auditory Cortex. Cerebral Cortex. 2021;31:1582–1596. 33136138 10.1093/cercor/bhaa311 PMC7869094

[36] Shannon RV , Zeng FG, Kamath V, Wygonski J, Ekelid M. Speech recognition with primarily temporal cues. Science. 1995;270:303–304. 7569981 10.1126/science.270.5234.303

[37] Davis MH , Johnsrude IS. Hearing speech sounds: Top-down influences on the interface between audition and speech perception. Hear Res. 2007;229(1–2):132–147. 17317056 10.1016/j.heares.2007.01.014

[38] Warren JD , Scott SK, Price CJ, Griffiths TD. Human brain mechanisms for the early analysis of voices. NeuroImage. 2006;31:1389–1397. 16540351 10.1016/j.neuroimage.2006.01.034

[39] Griffiths TD , Warren JD. The planum temporale as a computational hub. Trends Neurosci. 2002;25:348–353. 12079762 10.1016/s0166-2236(02)02191-4

[40] Obleser J , Wise RJS, Alex Dresner M, Scott SK. Functional integration across brain regions improves speech perception under adverse listening conditions. J Neurosci. 2007;27:2283–2289. 17329425 10.1523/JNEUROSCI.4663-06.2007 PMC6673469

[41] Hartwigsen G , Golombek T, Obleser J. Repetitive transcranial magnetic stimulation over left angular gyrus modulates the predictability gain in degraded speech comprehension. Cortex. 2015;68:100–110. 25444577 10.1016/j.cortex.2014.08.027

[42] Davis MH , Johnsrude IS. Hierarchical processing in spoken language comprehension. J Neurosci. 2003;23:3423–3431. 12716950 10.1523/JNEUROSCI.23-08-03423.2003 PMC6742313

[43] Heimbauer LA , Beran MJ, Owren MJ. A chimpanzee recognizes varied acoustical versions of sine-wave and noise-vocoded speech. Anim Cogn. 2021;24:843–854. 33555417 10.1007/s10071-021-01478-4

[44] Heimbauer Lisa A , Beran Michael J, Owren Michael J. A chimpanzee recognizes synthetic speech with significantly reduced acoustic cues to phonetic content. Curr Biol. 2011;21:1210–1214. 21723125 10.1016/j.cub.2011.06.007 PMC3143218

[45] Lahiff NJ , Slocombe KE, Taglialatela J, Dellwo V, Townsend SW. Degraded and computer-generated speech processing in a bonobo. Anim Cogn. 2022;25:1393–1398. 35595881 10.1007/s10071-022-01621-9 PMC9652166

[46] Nagarajan SS , Cheung SW, Bedenbaugh P, Beitel RE, Schreiner CE, Merzenich MM. Representation of spectral and temporal envelope of twitter vocalizations in common marmoset primary auditory cortex. J Neurophysiol. 2002;87:1723–1737. 11929894 10.1152/jn.00632.2001

[47] Joly O , Ramus F, Pressnitzer D, Vanduffel W, Orban GA. Interhemispheric differences in auditory processing revealed by fMRI in awake rhesus monkeys. Cereb Cortex. 2012;22:838–853. 21709178 10.1093/cercor/bhr150

[48] Cope TE , Sohoglu E, Sedley W, et al Evidence for causal top-down frontal contributions to predictive processes in speech perception. Nat Commun. 2017;8:2154. 29255275 10.1038/s41467-017-01958-7 PMC5735133

[49] Hardy CJ , Marshall CR, Golden HL, et al Hearing and dementia. J Neurol. 2016;263:2339–2354. 27372450 10.1007/s00415-016-8208-y PMC5065893

[50] Dubois B , Feldman HH, Jacova C, et al Advancing research diagnostic criteria for Alzheimer's disease: The IWG-2 criteria. The Lancet Neurology. 2014;13:614–629. 24849862 10.1016/S1474-4422(14)70090-0

[51] Gorno-Tempini ML , Hillis AE, Weintraub S, et al Classification of primary progressive aphasia and its variants. Neurology. 2011;76:1006–1014. 21325651 10.1212/WNL.0b013e31821103e6 PMC3059138

[52] Requena-Komuro M-C , Jiang J, Dobson L, et al Remote versus face-to-face neuropsychological testing for dementia research: A comparative study in patients with Alzheimer’s disease, patients with frontotemporal dementia, and healthy older individuals. BMJ Open. 2022;12:e064576. 10.1136/bmjopen-2022-064576 PMC9702828 36428012

[53] Schütt HH , Harmeling S, Macke JH, Wichmann FA. Painfree and accurate Bayesian estimation of psychometric functions for (potentially) overdispersed data. Vision Res. 2016;122:105–123. 27013261 10.1016/j.visres.2016.02.002

[54] Robin X , Turck N, Hainard A, et al pROC: An open-source package for R and S+ to analyze and compare ROC curves. BMC Bioinformatics. 2011;12:77. 21414208 10.1186/1471-2105-12-77 PMC3068975

[55] Hajian-Tilaki KO , Hanley JA, Joseph L, Collet J-P. A comparison of parametric and nonparametric approaches to ROC analysis of quantitative diagnostic tests. Med Decis Making. 1997;17:94–102. 8994156 10.1177/0272989X9701700111

[56] Malone IB , Leung KK, Clegg S, et al Accurate automatic estimation of total intracranial volume: A nuisance variable with less nuisance. Neuroimage. 2015;104:366–372. 25255942 10.1016/j.neuroimage.2014.09.034 PMC4265726

[57] Ridgway GR , Omar R, Ourselin S, Hill DLG, Warren JD, Fox NC. Issues with threshold masking in voxel-based morphometry of atrophied brains. NeuroImage. 2009;44:99–111. 18848632 10.1016/j.neuroimage.2008.08.045

[58] Hervais-Adelman AG , Carlyon RP, Johnsrude IS, Davis MH. Brain regions recruited for the effortful comprehension of noise-vocoded words. Lang Cogn Process. 2012;27(7–8):1145–1166.

[59] Scott SK , Rosen S, Lang H, Wise RJS. Neural correlates of intelligibility in speech investigated with noise vocoded speech—A positron emission tomography study. J Acoust Soc Am. 2006;120:1075–1083. 16938993 10.1121/1.2216725

[60] Gennari SP , Millman RE, Hymers M, Mattys SL. Anterior paracingulate and cingulate cortex mediates the effects of cognitive load on speech sound discrimination. NeuroImage. 2018;178:735–743. 29902588 10.1016/j.neuroimage.2018.06.035

[61] Desikan RS , Ségonne F, Fischl B, et al An automated labeling system for subdividing the human cerebral cortex on MRI scans into gyral based regions of interest. NeuroImage. 2006;31:968–980. 16530430 10.1016/j.neuroimage.2006.01.021

[62] Tomczak M , Tomczak E. The need to report effect size estimates revisited. An overview of some recommended measures of effect size. Trends in sport sciences. 2014;21:19–25.

[63] Miles J , Shevlin M. Applying regression and correlation: A guide for students and researchers. London, UK: Sage; 2001.

[64] Ohman EM , Granger CB, Harrington RA, Lee KL. Risk stratification and therapeutic decision making in acute coronary syndromes. JAMA. 2000;284:876–878. 10938178 10.1001/jama.284.7.876

[65] Carter JV , Pan J, Rai SN, Galandiuk S. ROC-ing along: Evaluation and interpretation of receiver operating characteristic curves. Surgery. 2016;159:1638–1645. 26962006 10.1016/j.surg.2015.12.029

[66] Wild CJ , Yusuf A, Wilson DE, Peelle JE, Davis MH, Johnsrude IS. Effortful listening: The processing of degraded speech Depends critically on attention. Journal of Neuroscience. 2012;32:14010–14021. 23035108 10.1523/JNEUROSCI.1528-12.2012 PMC6704770

[67] Warren JE , Wise RJ, Warren JD. Sounds do-able: Auditory-motor transformations and the posterior temporal plane. Trends Neurosci. 2005;28:636–643. 16216346 10.1016/j.tins.2005.09.010

[68] Shahin AJ , Bishop CW, Miller LM. Neural mechanisms for illusory filling-in of degraded speech. NeuroImage. 2009;44:1133–1143. 18977448 10.1016/j.neuroimage.2008.09.045 PMC2653101

[69] Obleser J , Kotz SA. Expectancy constraints in degraded speech modulate the language comprehension network. Cereb Cortex. 2010;20:633–640. 19561061 10.1093/cercor/bhp128

[70] Golestani N , Hervais-Adelman A, Obleser J, Scott SK. Semantic versus perceptual interactions in neural processing of speech-in-noise. NeuroImage. 2013;79:52–61. 23624171 10.1016/j.neuroimage.2013.04.049

[71] Lombardi J , Mayer B, Semler E, et al Quantifying progression in primary progressive aphasia with structural neuroimaging. Alzheimers Dement. 2021;17:1595–1609. 33787063 10.1002/alz.12323

[72] Ruksenaite J , Volkmer A, Jiang J, et al Primary progressive aphasia: Toward a pathophysiological synthesis. Curr Neurol Neurosci Rep. 2021;21:7. 33543347 10.1007/s11910-021-01097-z PMC7861583

[73] Bejanin A , Schonhaut DR, La Joie R, et al Tau pathology and neurodegeneration contribute to cognitive impairment in Alzheimer’s disease. Brain. 2017;140:3286–3300. 29053874 10.1093/brain/awx243 PMC5841139

[74] Giannini LAA , Irwin DJ, McMillan CT, et al Clinical marker for Alzheimer disease pathology in logopenic primary progressive aphasia. Neurology. 2017;88:2276–2284. 28515265 10.1212/WNL.0000000000004034 PMC5567322

[75] Johnson JCS , Jiang J, Bond RL, et al Impaired phonemic discrimination in logopenic variant primary progressive aphasia. Ann Clin Transl Neurol. 2020;7:1252–1257. 32558373 10.1002/acn3.51101 PMC7359108

[76] Shenhav A , Botvinick Matthew M, Cohen Jonathan D. The expected value of control: An integrative theory of anterior cingulate Cortex function. Neuron. 2013;79:217–240. 23889930 10.1016/j.neuron.2013.07.007 PMC3767969

[77] Abutalebi J , Della Rosa PA, Green DW, et al Bilingualism tunes the anterior cingulate Cortex for conflict monitoring. Cerebral Cortex. 2012;22:2076–2086. 22038906 10.1093/cercor/bhr287

[78] Goll JC , Kim LG, Hailstone JC, et al Auditory object cognition in dementia. Neuropsychologia. 2011;49:2755–2765. 21689671 10.1016/j.neuropsychologia.2011.06.004 PMC3202629

[79] Stalpaert J , Miatton M, Sieben A, Langenhove TV, Pv M, Letter MD. The electrophysiological correlates of phoneme perception in primary progressive aphasia: A preliminary case series. Front Hum Neurosci. 2021;15:618549. 34149376 10.3389/fnhum.2021.618549 PMC8206281

[80] Goll JC , Crutch SJ, Loo JH, et al Non-verbal sound processing in the primary progressive aphasias. Brain. 2010;133(Pt 1):272–285. 19797352 10.1093/brain/awp235 PMC2801322

[81] Grube M , Bruffaerts R, Schaeverbeke J, et al Core auditory processing deficits in primary progressive aphasia. Brain. 2016;139(Pt 6):1817–1829. 27060523 10.1093/brain/aww067 PMC4892752

[82] Meister H , Schreitmüller S, Grugel L, Beutner D, Walger M, Meister I. Examining speech perception in noise and cognitive functions in the elderly. Am J Audiol. 2013;22:310–312. 24018577 10.1044/1059-0889(2012/12-0067)

[83] Millman RE , Mattys SL. Auditory verbal working memory as a predictor of speech perception in modulated maskers in listeners with normal hearing. J Speech Lang Hear Res. 2017;60:1236–1245. 28492912 10.1044/2017_JSLHR-S-16-0105

[84] Hardy CJD , Frost C, Sivasathiaseelan H, et al Findings of impaired hearing in patients with nonfluent/agrammatic variant primary progressive aphasia. JAMA Neurol. 2019;76:607–611. 30742208 10.1001/jamaneurol.2018.4799 PMC6515576

[85] Peelle JE . Listening effort: How the cognitive consequences of acoustic challenge are reflected in brain and behavior. Ear Hear. 2018;39:204–214. 28938250 10.1097/AUD.0000000000000494 PMC5821557

[86] Davis MH , Johnsrude IS, Hervais-Adelman A, Taylor K, McGettigan C. Lexical information drives perceptual learning of distorted speech: Evidence from the comprehension of noise-vocoded sentences. J Exp Psychol Gen. 2005;134:222–241. 15869347 10.1037/0096-3445.134.2.222

[87] Sohoglu E , Davis MH. Perceptual learning of degraded speech by minimizing prediction error. Proc Natl Acad Sci U S A. 2016;113:E1747–E1756. 26957596 10.1073/pnas.1523266113 PMC4812728

[88] Hervais-Adelman A , Davis MH, Johnsrude IS, Carlyon RP. Perceptual learning of noise vocoded words: Effects of feedback and lexicality. Journal of Experimental Psychology: Human Perception and Performance. 2008;34:460–474. 18377182 10.1037/0096-1523.34.2.460

[89] Alegret M , Boada-Rovira M, Vinyes-Junqué G, et al Detection of visuoperceptual deficits in preclinical and mild Alzheimer's disease. J Clin Exp Neuropsychol. 2009;31:860–867. 19142775 10.1080/13803390802595568 PMC2834652

[90] Utoomprurkporn N , Stott J, Costafreda SG, Bamiou DE. Lack of association between audiogram and hearing disability measures in mild cognitive impairment and dementia: What audiogram does not tell you. Healthcare (Basel). 2021;9:769. 34203041 10.3390/healthcare9060769 PMC8234005

[91] Johnson JCS . Hearing impairment in dementia: Defining deficits and assessing impact. PhD thesis. University College London; 2021.

