# Supplementary material for: Comprehension of acoustically degraded speech in Alzheimer’s disease and primary progressive aphasia
Source: Brain. 2023 May 15;146(10):4065–76. doi: 10.1093/brain/awad163 (PMC10545509; doi:10.1093/brain/awad163)
Supplement: awad163_Supplementary_Data [file awad163_supplementary_data.zip › brain-2022-02323-File008.pdf]

SUPPLEMENTARY MATERIAL: Comprehension of acoustically degraded speech in Alzheimer's disease and primary progressive aphasia, by J Jiang et al

Assessment of peripheral hearing function

A standard clinical audiometer protocol was used to assess participants' peripheral hearing of pure tones at set frequencies [92]. Using a GSI Audiostar Pro™ audiometer (https://www.guymark.com/product-information/hearing-assessment/audiometers/clinical/gsi-audiostar-pro) and calibrated GSI Audiostar Pro headphones with noise-reducing ear-cups, steady tones of 250, 500, 1000, 2000, 4000 and 8000Hz were presented separately to each of the participant's ears. At each frequency, the participant indicated (verbally or by gesture) when they first heard a noise. The decibel hearing level was set typically at 50 dB, with decreases of 10 dB if they could hear the tone, and increases of 5dB if they could not.. For each participant, a composite peripheral hearing score was created by calculating the mean threshold across all frequencies in the best ear.

The PALPA-3 phoneme discrimination test

The Psycholinguistic Assessment of Language Processing in Aphasia (PALPA) battery includes a "minimal pairs" test (PALPA-3) which assesses discrimination of clear speech phonemes differing on a single acoustic characteristic.[95] On each trial, participants were asked to indicate which of two written words matches the spoken monosyllabic word (e.g., spoken "leave" – written response choices: (1) leave and (2) leaf). We adopted a subset of 36 trials from the full PALPA-3 test (72-items).

Synthesis of noise-vocoded stimuli

Raw speech recordings were noise-vocoded using a script written by Chris Darwin: http://www.lifesci.sussex.ac.uk/home/Chris_Darwin/Praatscripts/Shannon. The logspace function in Matlab was used to calculate log spacing between 50 and 8000Hz, corresponded to logarithmically-spaced frequency bands that served as the basis for the noise-vocoding algorithm. The algorithm was run iteratively to generate speech stimuli with between 1 and 24 frequency bands ('channels').

Average (rms) stimulus intensity was constant for all stimuli, as fixed in Matlab. All stimuli were windowed with 20ms onset-offset temporal ramps to prevent click artefacts.

Remote testing procedure

Thirty participants (four healthy controls, nine patients with Alzheimer's disease (AD), seven with logopenic variant primary progressive aphasia (lvPPA), six with nonfluent/agrammatic variant primary progressive aphasia (nfvPPA) and four with semantic variant primary progressive aphasia (svPPA)) were assessed remotely via a video link, due to the COVID-19 pandemic. We have detailed the design and implementation of our remote neuropsychological assessment protocol elsewhere [52].

An initial session was conducted via Zoom to accustom participants to the remote testing format, check the screen and sound sharing options on Zoom, and that the quality of the participant's internet connection was acceptable. Participants were permitted to use their preferred technology interface (computers/tablets; smartphones were not allowed, to ensure good screen visibility). Remote assessments were scheduled to ensure testing could be completed in a quiet environment with minimal distractions, and the device volume was set to a comfortable level by each participant or their caregiver. The experiment was implemented onto Labvanced for administration.[93] Most participants listened in free field, over their device speakers (only a few used headphones); of the seven healthy control participants who performed the experiment both in person and remotely, only one used headphones for the remote session.

A further audibility check was used for each participant through the Bamford-Kowal-Bench [94] list. The spoken BKB sentences were delivered online using Labvanced [93]; a perfect score of the final 3 items on the test was required to go on to the remote testing session proper (this allowed each participant and/or caregiver to manually adjust the volume to a comfortable level for clear audibility). Most participants (97%) performed at ceiling; none was rejected based on their BKB performance.

Each patient's caregiver was required to be available during each research session in case of any problems using the equipment. In practice, however, no major technological issues arose during the remote testing sessions.

The remote experimental procedure was otherwise identical to the in-person procedure.

Performance profiles in seven healthy control participants who performed the experiment both in person (via headphones) and remotely (in free-field) were very similar (see Figure S6), justifying combining participants tested in person and remotely in the main analysis.

Experimental behavioural data results: parallel analyses after outlier removal

A separate analysis was conducted without the two upper bound outliers (one nfvPPA and one lvPPA) of the speech intelligibility threshold (>97.5 quantile). There was a significant main effect of diagnostic group on noise-vocoded speech intelligibility threshold ($H(4) = 37.74$, $p<0.001$). In post-hoc pairwise group comparisons versus healthy controls, mean intelligibility threshold was significantly elevated in all patient groups: in the lvPPA ($z=4.11$, $p<0.001$), nfvPPA ($z=3.60$, $p<0.001$), AD ($z=5.22$, $p<0.001$) and svPPA ($z=2.29$, $p=0.02$) groups. Comparing patient groups, intelligibility threshold was significantly elevated in the AD ($z=2.13$, $p=0.03$) and lvPPA ($z=2.01$, $p=0.04$) groups compared with the svPPA group. There was no significant effect of diagnostic group on the slope of the psychometric function ($p=0.419$). There was a significant main effect of diagnostic group on the lapse rate, lambda ($H(4) = 19.98$, $p<0.001$). In post-hoc pairwise group comparisons versus healthy controls, there was a significantly higher lapse rate (more errors made at maximum performance) in all patient groups: in the lvPPA ($z=3.01$, $p=0.003$), AD ($z=2.60$, $p=0.009$), nfvPPA ($z=3.57$, $p<0.001$), and svPPA ($z=2.30$, $p=0.02$) groups. There were no significant differences between patient groups for lapse rate. There was a significant main effect of diagnostic group on the guess rate, gamma ($H(4) = 15.80$, $p=0.003$). In post-hoc pairwise group comparisons, there was a significantly higher gamma rate (more correct answers made at minimum performance) in the healthy control group than other patient groups ($p<0.05$). There was no significant effect of diagnostic group on on *eta* (overdispersion of the data) of the psychometric function ($p=0.116$).

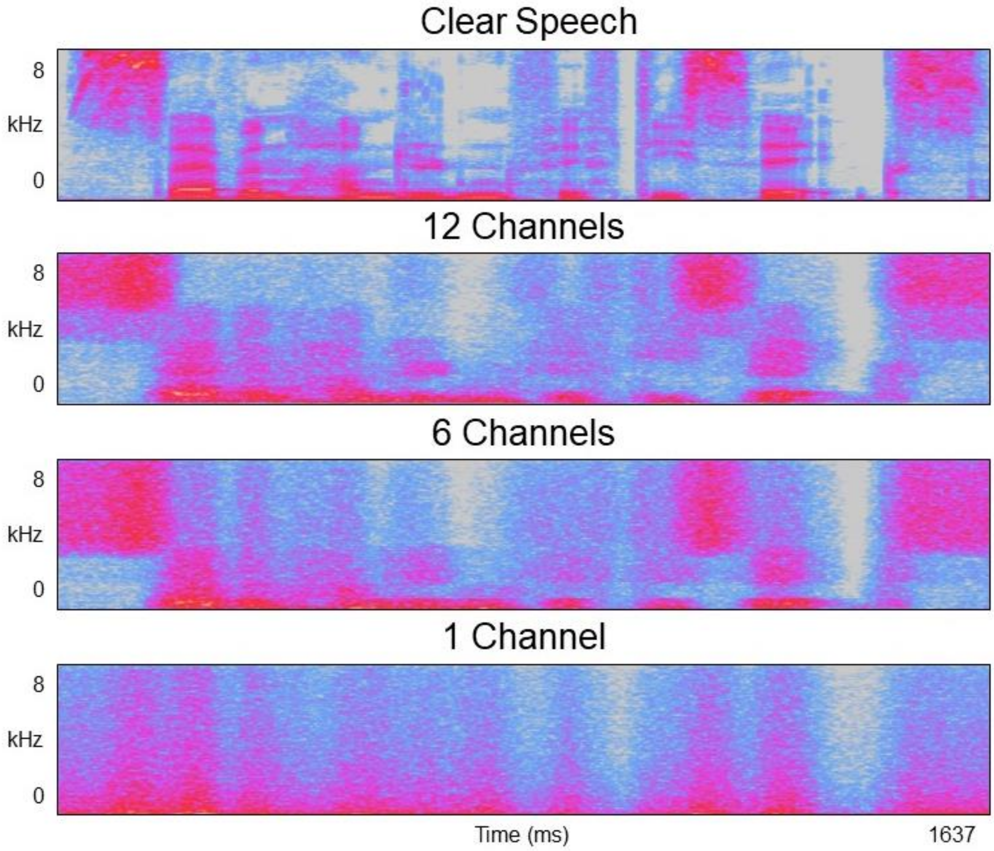

Figure S1. Spectrograms of clear (natural) speech and noise-vocoded speech resampled with differing numbers of discrete frequency bands (12 channels, 6 channels and 1 channel). The verbal message in each case is the spoken number 'seven hundred and fifty-six'. The spectrogram provides a visual indication of how the energy in different frequency bands of the speech signal (plotted on y axis) changes over time (plotted on x-axis). Reducing the number of channels (frequency bands) reduces progressively the amount of spectrotemporal fine structure in the speech signal. Example stimuli are available in Supplementary Materials online.

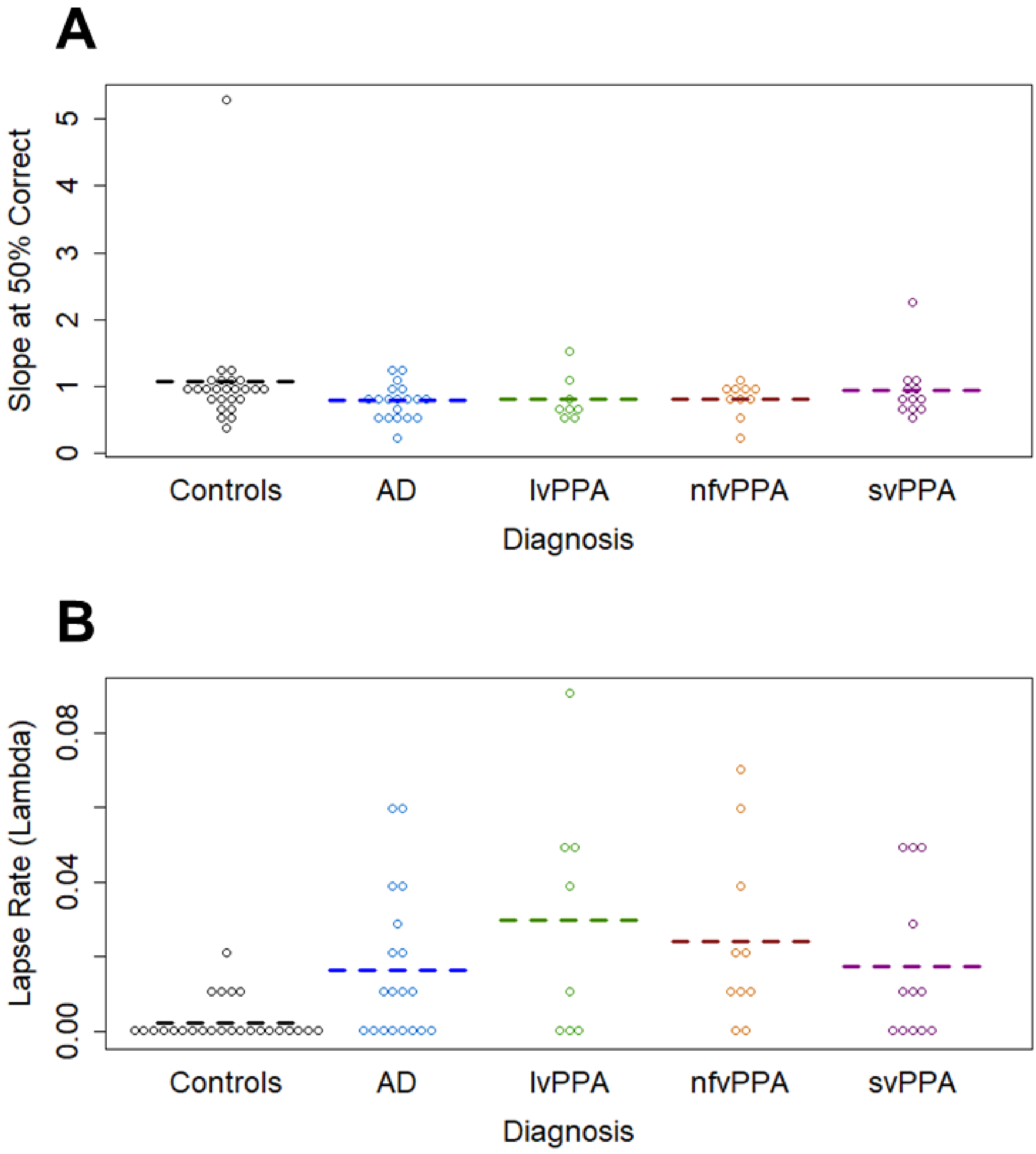

Figure S2. Beeswarm plots of individual participants' psychometric parameters for comprehension of noise-vocoded speech within each diagnostic group. Dashed lines represent the mean for each group. **A,** Slope values for each participant at 50% intelligibility. **B,** Lapse rate (lambda) showing the errors that occurred at the maximum performance asymptote. AD, patient group with typical Alzheimer's disease; Control, healthy older control group; lvPPA, patient group with logopenic variant primary progressive aphasia; nfvPPA, patient group with nonfluent variant primary progressive aphasia; svPPA, patient group with semantic variant primary progressive aphasia.

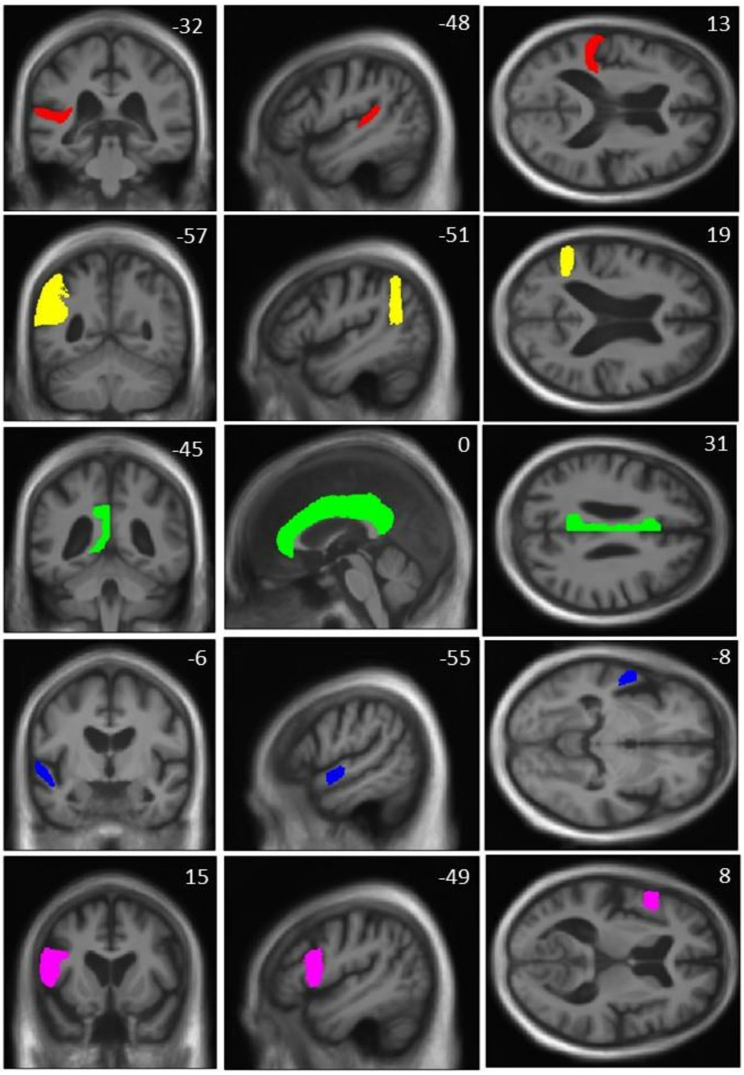

Figure S3. Representative sections of neuroanatomical regions in the left cerebral hemisphere that were used for multiple voxel-wise comparisons correction in region-of-interest analyses (see text). Regions are rendered on coronal (left), sagittal (middle) and axial (right) sections of the mean normalised brain template for the patient cohort; MNI coordinates of the plane of each section are shown. The neuroanatomical regions comprise left planum temporale (red), left angular gyrus (yellow), left cingulate gyrus (green), left anterior superior temporal gyrus (blue), and left inferior frontal gyrus (purple).

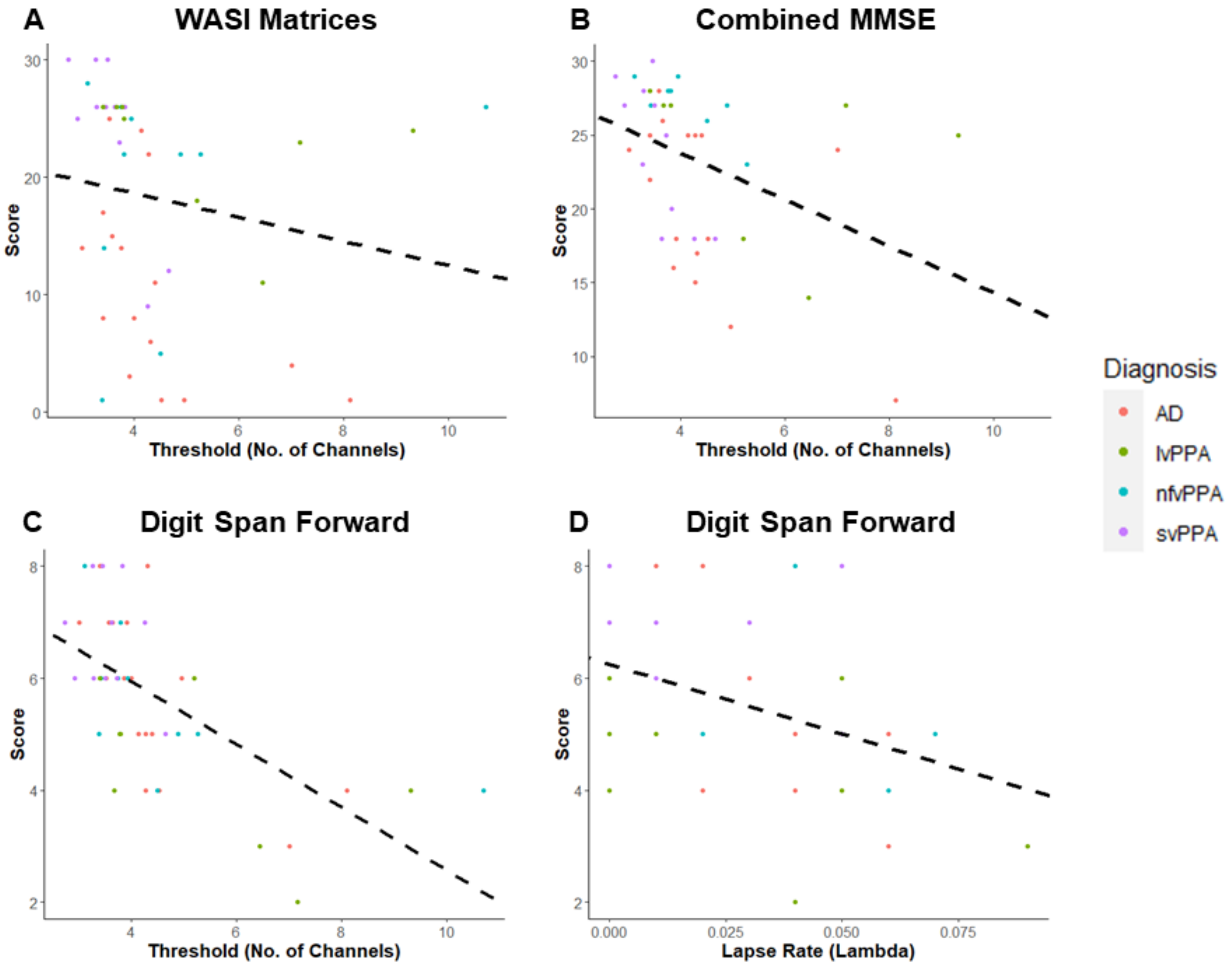

Figure S4. Correlation plots for comprehension of noise-vocoded speech in each participant group. Dots represent individual participants, colour-coded by diagnostic group. The black dashed regression line is fitted across the combined patient cohort. **A,** Correlation plot between the speech intelligibility threshold and WASI matrix performance (r=-0.46, p=0.001). **B,** Correlation plot between the speech intelligibility threshold and combined MMSE performance (r=-0.53, p<0.001). **C,** Correlation plot between speech intelligibility threshold and forward digit span performance (r=-0.63, p<0.001). **D,** Correlation plot between the lapse rate (lambda) and forward digit span performance (r=-0.34, p=0.018). AD, patient group with typical Alzheimer's disease; lvPPA, patient group with logopenic variant primary progressive aphasia; nfvPPA, patient group with nonfluent variant primary progressive aphasia; svPPA, patient group with semantic variant primary progressive aphasia.

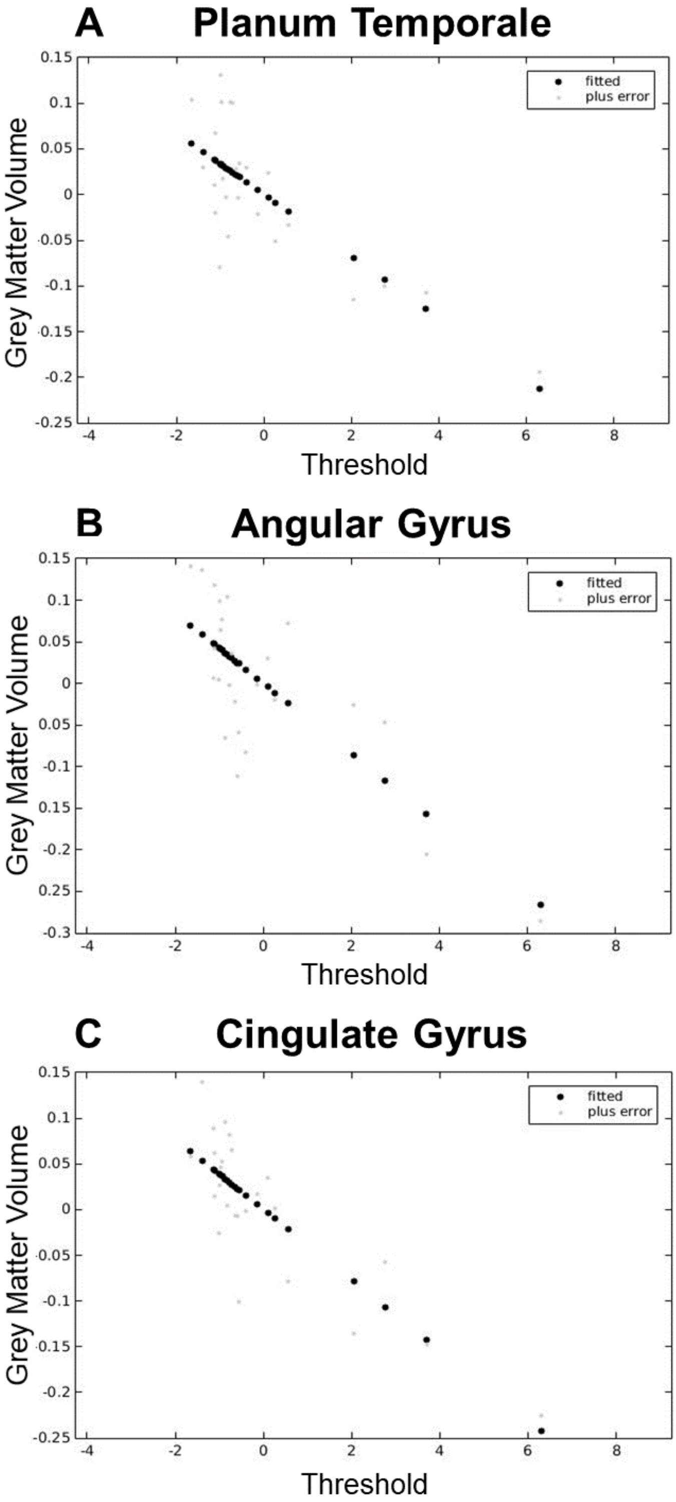

Figure S5. Correlation plots for speech intelligibility threshold and significant peak regions, details in Table 3. The threshold measure reported here is mean-centred. **A,** Correlation plot between the speech intelligibility threshold and the left planum temporale at peak [-48, -31, 6] (t=4.65, p=0.019). **B,** Correlation plot between the speech intelligibility threshold and the left angular gyrus at peak [-51, -61, 16] (t=4.51, p=0.037). **C,** Correlation plot between the speech intelligibility threshold and the left cingulate gyrus at peak [-1, 38, -5] (t=5.68, p=0.012).

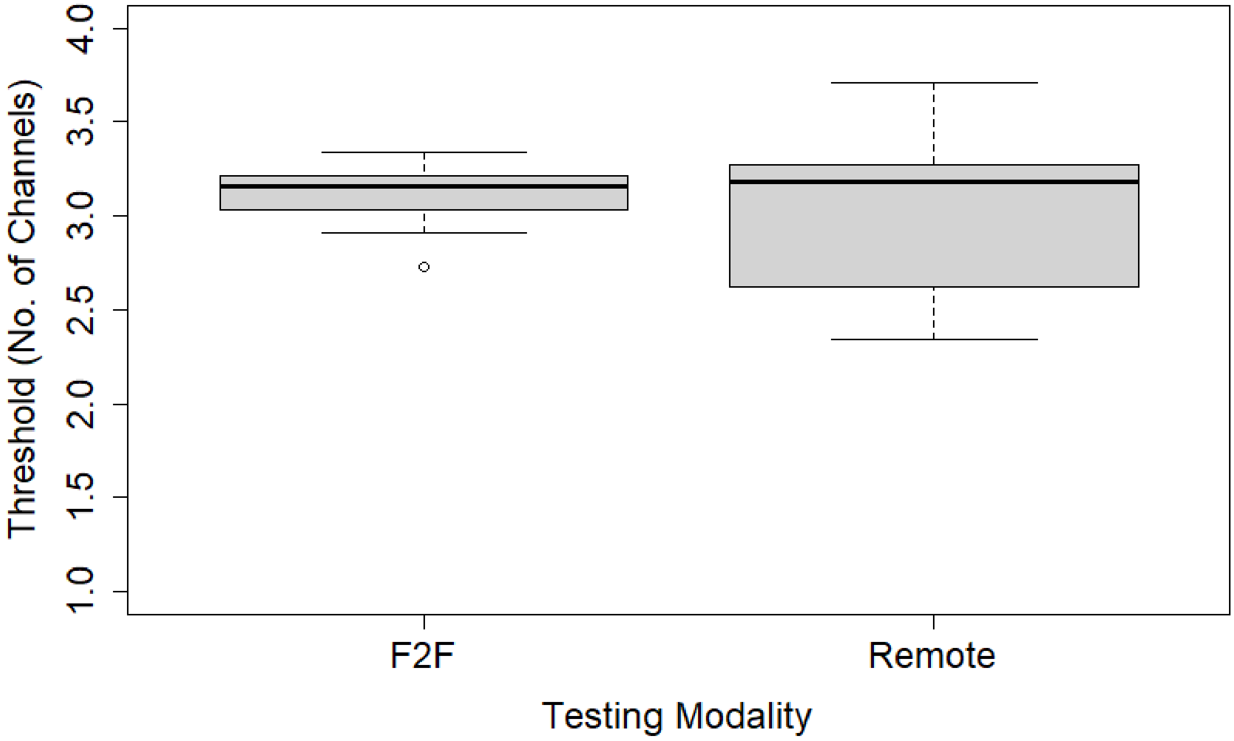

Figure S6. Comparison of healthy older controls' performance on comprehension of noise-vocoded speech for in-person vs remote testing sessions. Seven healthy older control participants performed the noise-vocoded spoken number identification task both face-to-face (F2F) at the research centre and remotely in their home environments, approximately 20 months later. Box plots show the mean and standard deviation (whiskers indicate the full range) of speech intelligibility thresholds in each session. Threshold differences between the two sessions were non-significant (F2F mean = 3.105; Remote mean = 3.003; p=0.69).

References

92. Audiology BSo. Recommended Procedure: Pure-tone air-conduction and bone-conduction threshold audiometry with and without masking. 2018.
93. Finger H, Goeke C, Diekamp D, Standvoß K, König P. Labvanced: A unified JavaScript framework for online studies. 2017.
94. Bench J, Kowal Å, Bamford J. The bkb (bamford-kowal-bench) sentence lists for partially-hearing children. Br J Audiol. 1979;13:108-112.
95. Kay J, Lesser R, Coltheart M. Psycholinguistic assessments of language processing in aphasia. UK: Lawrence Erlbaum Associates;1992.
